# Crystal structures of three hexakis­(fluoroar­yloxy)cyclo­triphosphazenes

**DOI:** 10.1107/S2056989019012933

**Published:** 2019-09-27

**Authors:** Hemant P. Yennawar, Andrew R. Hess, Harry R. Allcock

**Affiliations:** a Pennsylvania State University, Department of Biochemistry and Molecular Biology, 108 Althouse Laboratory, University Park, PA 16802, USA; b Pennsylvania State University, Department of Chemistry, 118 Chemistry Building, University Park, PA 16802, USA

**Keywords:** organophosphazine, hexa­kis­(penta­fluoro­phen­oxy)cyclo­triphosphazene, hexa­kis­(4-tri­fluoro­methyl­phen­oxy)cyclo­triphosphazene, hexa­kis­(3,5-bis tri­fluoro­methyl­phen­oxy)cyclo­triphosphazene, envelope pucker, C—H(π)⋯F inter­actions, crystal structure

## Abstract

The syntheses and crystal structures of three cyclo­triphosphazenes, all with fluorinated ar­yloxy side groups that generate different steric characteristics, *viz*. hexa­kis­(penta­fluoro­phen­oxy)cyclo­triphosphazene, N_3_P_3_(OC_6_F_5_)_6_, **1**, hexa­kis­[4-(tri­fluoro­methyl)­phen­oxy]cyclo­triphosphazene, N_3_P_3_[OC_6_H_4_(CF_3_)]_6_, **2** and hexa­kis­[3,5-bis(­tri­fluoro­methyl)­phen­oxy]cyclo­triphosphazene, N_3_P_3_[OC_6_H_3_(CF_3_)_2_]_6_ **3**, are reported. Specifically, each phospho­rus atom bears either two penta­fluoro­phen­oxy, 4-tri­fluoro­methyl­phen­oxy, or 3,5-tri­fluoro­methyl­phen­oxy groups. The central six-membered phosphazene rings display envelope pucker conformations in each case, albeit to varying degrees. The maximum displacement of the ‘flap atom’ from the plane through the other ring atoms [0.308 (5) Å] is seen in **1**, in a mol­ecule that is devoid of hydrogen atoms and which exhibits a ‘wind-swept’ look with all the aromatic rings displaced in the same direction. In **3** an intra­molecular C—H(aromatic)⋯F inter­action is observed. All the –CF_3_ groups in **2** and **3** exhibit positional disorder over two rotated orientations in close to statistical ratios. The extended structures of **2** and **3** are consolidated by C—H⋯F inter­actions of two kinds: (*a*) linear chains, and (*b*) cyclic between mol­ecules related by inversion centers. In both **1** and **3**, one of the six substituted phenyl rings has a parallel-displaced aromatic π–π stacking inter­action with its respective symmetry mate with slippage values of 2.2 Å in **1** and 1.0 Å in **3**. None of the structures reported here have solvent voids that could lead to clathrate formation.

## Abstract 2

The syntheses and crystal structures of three cyclo­triphosphazenes, all with fluorinated ar­yloxy side groups that generate different steric characteristics are reported.

## Chemical context   

Cyclic organophosphazenes have a long history as representatives of inorganic heterocyclic rings, and are also the focus of arguments about reactivity and pseudoaromaticity in inorganic systems (Allcock, 1972 ▸; Steudel, 1992 ▸). In our research program these compounds also serve another purpose – as synthesis and structural models for linear high polymeric organophosphazenes. These cyclic small mol­ecules provide preliminary information related to intra- and inter­molecular side-group inter­actions, which affect many polymer properties.

As part of our ongoing work in this area, the three cyclo­triphosphazenes hexa­kis­(penta­fluoro­phen­oxy)cyclo­tri­phos­phazene N_3_P_3_(OC_6_F_5_)_6_ (**1**), hexa­kis­[4-(tri­fluoro­methyl)­phenoxy]cyclo­triphosphazene N_3_P_3_[OC_6_H_4_(CF_3_)]_6_ (**2**) and hexa­kis­[3,5-bis(tri­fluoro­methyl­phen­oxy]cyclo­triphosphazene; N_3_P_3_[OC_6_H_3_(CF_3_)_2_]_6_ (**3**) were synthesized by the reactions of hexa­chloro­cyclo­triphosphazene with the appropriate sodium fluoro-aryl­oxides in THF or dioxane solvent. Forcing reaction conditions (boiling dioxane) were required for complete chlorine replacement in the case of the penta­fluoro­phen­oxy derivative (**1**) presumably due to steric hindrance. The three compounds were characterized by NMR spectroscopy in addition to x-ray crystallography.

The structure of a fourth, related cyclo­triphosphazene with six *para*-fluoro­phen­oxy groups, was reported by other investigators (Wahl *et al.*, 2012 ▸) and was independently verified by us. The non-fluorinated hexa­(phen­oxy)cyclo­triphosphazene tetra­hydro­furan solvate x-ray structure was also described earlier (Dietrich *et al.*, 2000 ▸).
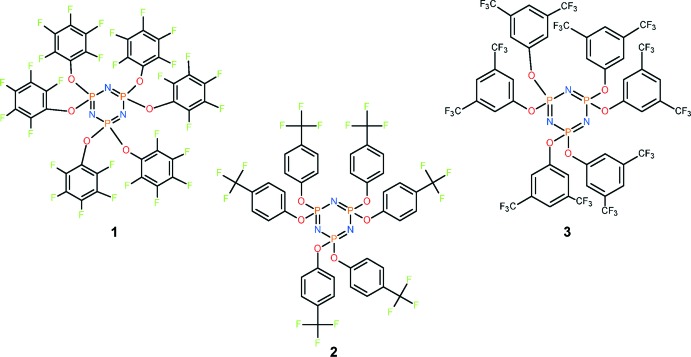


## Structural commentary   

**Figure 1:**
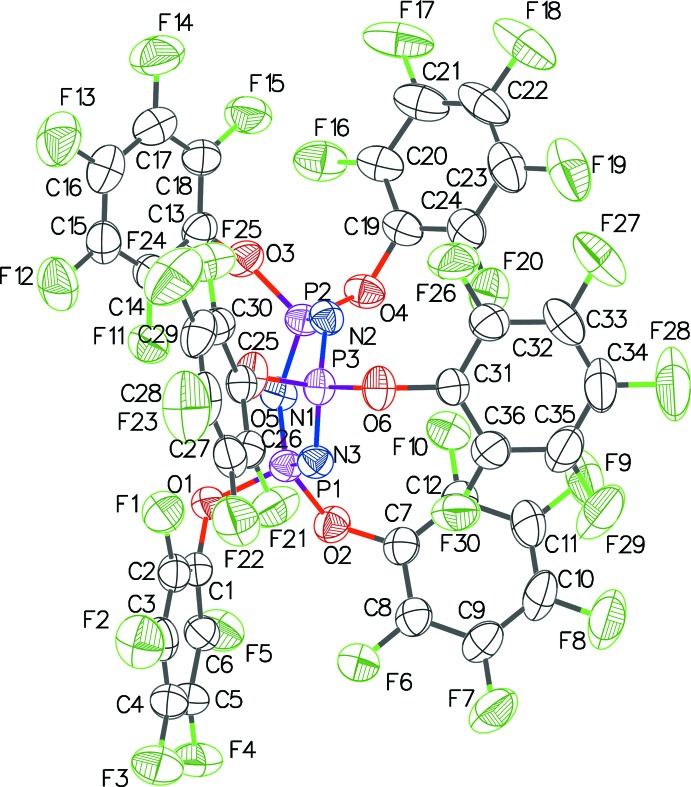
The mol­ecular structure of **1** with displacement ellipsoids drawn at the 50% probability level.

The structures of **1**, **2** and **3** (Figs. 1 ▸, 2 ▸ and 3 ▸) presented in this report have the hexa­phen­oxy-cyclo­triphosphazene moiety as the common core of the mol­ecule, and differ only in the substitutions on the phenyl rings. The cyclo­triphosphazene ring in these structures exhibit varying degree of envelope pucker. The maximum pucker is seen in **1** wherein the displacement of atom P3 from the plane defined by the other five atoms of the ring (each member atom less than 0.05 Å from the plane) is 0.308 (5) Å. A similar calculation in **2** shows atom P2 displaced by 0.232 (4) Å, and in **3** atom N3 is displaced the least, only by 0.205 (4) Å. In earlier structure reports: [(NPCl_2_)_3_: Bullen, 1971 ▸; (NPF_2_)_3_: Singh *et al.*, 2000 ▸; (NPPh_2_)_3_·THF: Dietrich *et al.*, 2000 ▸], the cyclo­triphosphazene ring was always planar, *i.e.* no puckering was seen.

**Figure 4:**
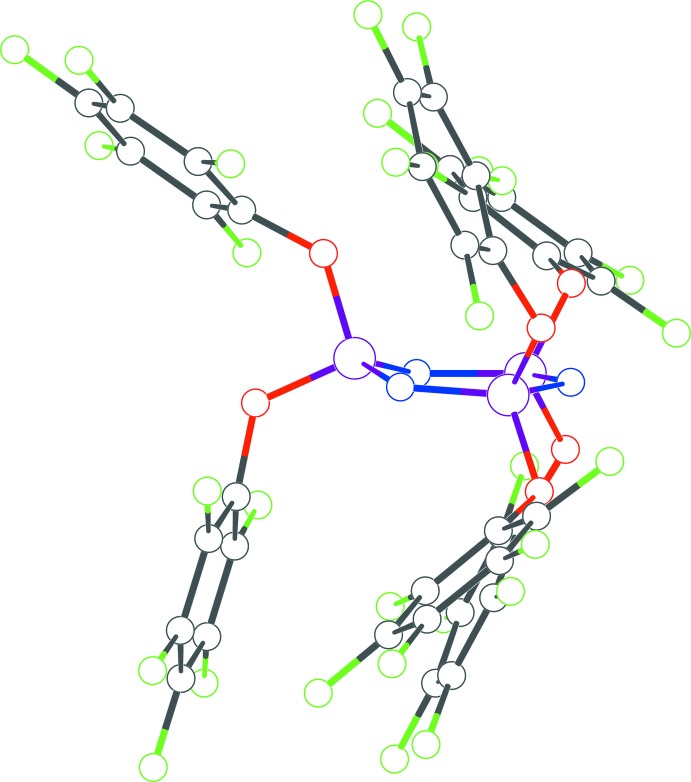
The mol­ecule of **1** showing all six penta­flourophen­oxy rings leaning to one side with respect to the central cyclo­triphosphazene moiety.

The mol­ecular structure of **1** has a ‘wind-swept’ appearance (Fig. 4 ▸) with all six penta­fluoro­phenyl rings seemingly pushed in one direction with respect to the cyclo­triphosphazene ring. In all the structures here, a pair of aryl­oxy groups is attached to each of the three phospho­rous atoms of each mol­ecule. Comparing the orientation of the rings within each pair, in **1** they are almost orthogonal to each other with the three dihedral angles being 72.3 (2), 76.1 (2) and 80.3 (2)°; in **2** they are between parallel and orthogonal with dihedral angles of 27.3 (2), 33.2 (2) and 62.6 (2)°, and in **3** the dihedral angles cover the widest range: 30.2 (2), 45.1 (2) and 82.4 (2)°. The tri­fluoro methyl groups in **2** and **3** are all positionally disordered. A C10—H10⋯F26 intra­molecular inter­action is observed in **3** [H⋯F = 2.58, C⋯F = 3.478 (4) Å, C—H⋯F = 163°].

## Supra­molecular features   

**Figure 5:**
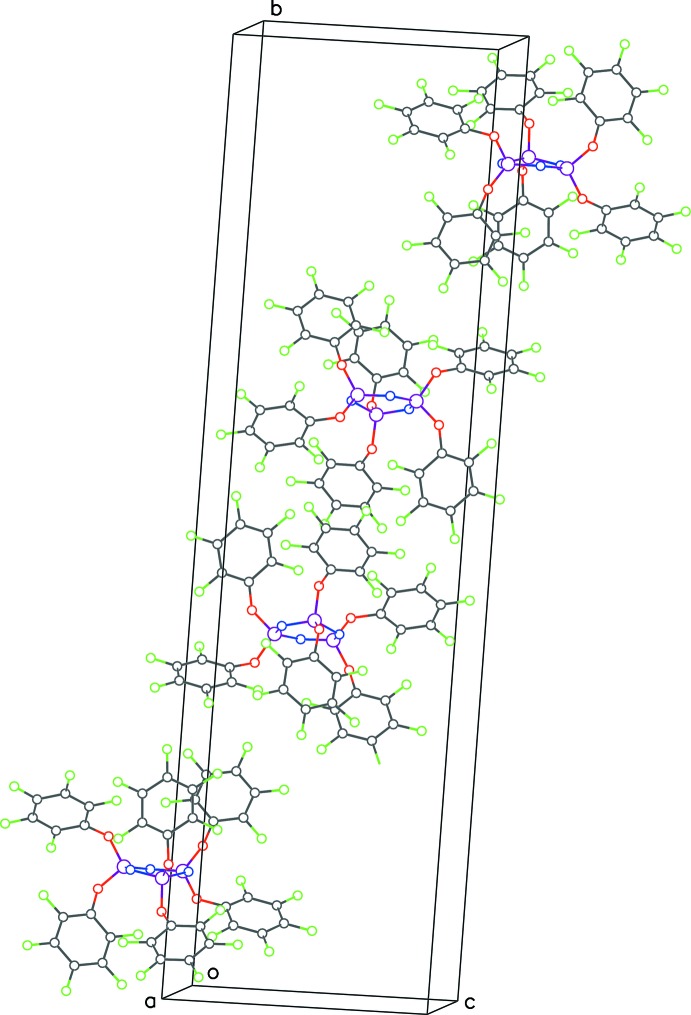
Packing diagram for **1** viewed down *b*-axis direction.

**Figure 6:**
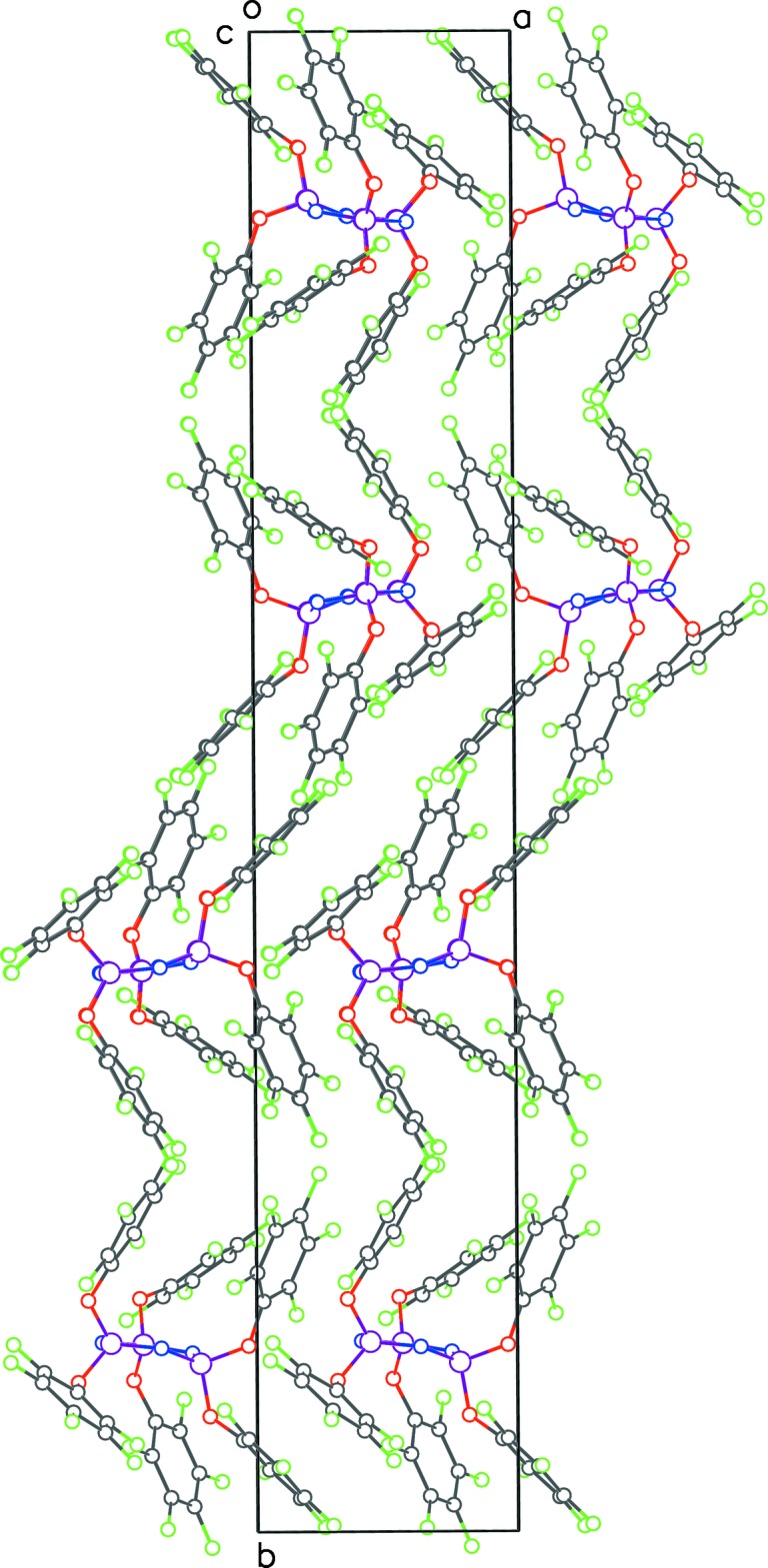
Unit-cell contents of **1** viewed down the *c-*axis direction, showing the nestling of the mol­ecules. The top two rows face opposite to the bottom two rows.

With no hydrogen atom in the mol­ecule of **1**, hydrogen bonding is not feasible in that structure (see packing diagram in Fig. 5 ▸). The mol­ecules nestle along the *a*-axis direction, with two adjacent rows facing in one direction and the other two in the opposite direction (Fig. 6 ▸). A weak parallel-displaced π–π ring inter­action is observed between rings related by inversion symmetry [C25–C30, centroid–centroid distance = 4.030 (2) Å, slippage = 2.22 Å].

**Figure 7:**
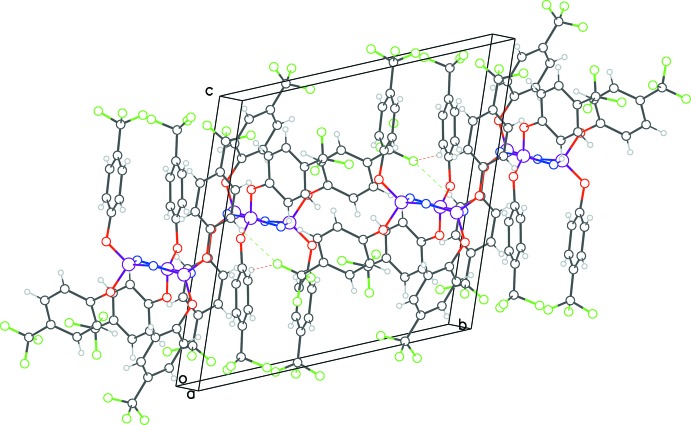
Packing diagram for **2** viewed along the *a*-axis direction. The dashed lines show inter­molecular C—H⋯F inter­actions – red ones for cyclic inter­actions between mol­ecules straddling the inversion center and the green for inter­actions generating continuous chains along the *b*-axis direction.

In the extended structure of **2**, the mol­ecules form chains linked by C—H(π)⋯F type hydrogen bonds (Table 1 ▸, Fig. 7 ▸), along the *b-*axis direction. Pairs of centrosymmetrically related mol­ecules inter­act with cyclic hydrogen bonds. No π–π ring inter­actions are seen in this structure.

**Figure 8:**
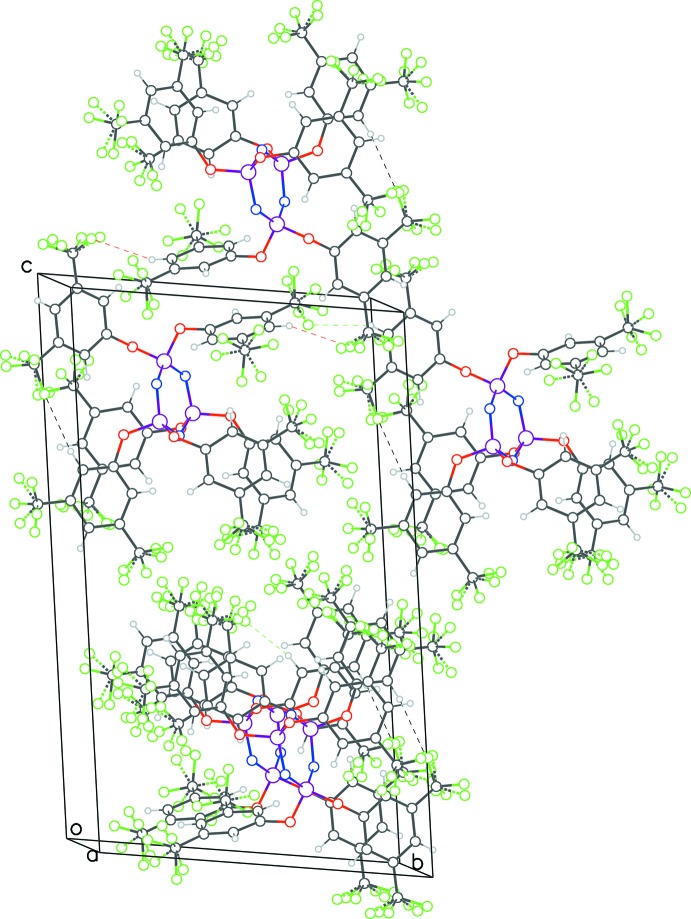
Packing diagram for **3** viewed along the *a*-axis direction. The black dashed lines show intra­molecular C—H⋯F inter­actions, while the red ones show cyclic inter­action between mol­ecules straddling the inversion center. The green lines are for inter­actions leading to continuous chains along *a*- and *b*-axis directions.

The packing diagram of **3** shows mol­ecules linked by C—H(π)⋯F hydrogen bonds (Table 2 ▸, Fig. 8 ▸), forming chains propagating along the *a* and *b*-axis directions. In addition, pairs of mol­ecules related by inversion centers have cyclic hydrogen bonding between them. A parallel π–π stacking inter­action is observed between one of the six phen­oxy rings and its symmetry mate [C41–C46, centroid–centroid distance = 3.646 (2) Å, slippage 1.013 Å].

Solvent-accessible voids are not present in any of the structures reported here. The solvent inclusion or lack thereof in the crystals of cyclo­triphosphazene compounds has been discussed by Wahl *et al.* (2016 ▸).

## Database survey   

Earlier, we reported the crystal structures of a number of cyclo­triphosphazenes with spiro­cyclic ar­yloxy side groups (Lee *et al.*, 2010 ▸). These are clathrate systems that trap hydro­carbon mol­ecules in the cage or tunnel structures. However, the structures in the present study, as well as a series of polymorph structures of hexa­kis­(4-fluoro­phen­oxy) cyclo­triphosphazene reported by Wahl *et al.* (2016 ▸) have no solvent-accessible voids. Various cyclo­triphosphazene structures have been reported in the literature: [(NPCl_2_)_3_, CSD refcode KAGKUY: Bullen, 1971 ▸; (NPF_2_)_3_, VARYES02: Singh *et al.*, 2000 ▸; (NPPh_2_)_3_·THF, GUHPII: Dietrich *et al.*, 2000 ▸]. For a review of the expanding field of polyphosphazene high polymers, see: Allcock (2016 ▸).

## Synthesis and crystallization   

**Synthesis of hexa­kis­(penta­fluoro­phen­oxy)cyclo­triphos­pha­zene (1):**

Sodium penta­fluoro­phenoxide was prepared by the treatment of penta­fluoro­phenol (15.88 g, 86 mmol) with a suspension of NaH 60% dispersion in mineral oil (3.10 g, 78 mmol) in 50 ml of dioxane. The penta­fluoro­phenoxide was added to a stirred solution of hexa­chloro­cyclo­triphosphazene (3.00 g, 8.6 mmol) and the mixture was heated at reflux for 3 d. Dioxane was removed from the mixture by rotary evaporation and the residue was dissolved in 100 ml di­chloro­methane. The solution was extracted with 3 × 100 ml of deionized water, dried over MgSO_4_, and concentrated to ∼10 ml by rotary evaporation. A small amount of hexa­nes was added to the concentrated solution and it was chilled to 273 K *via* an ice bath to yield colorless blocks of **1**, which were filtered and rinsed with cold hexa­nes then dried under vacuum.

**Synthesis of hexa­kis­(4-tri­fluoro­methyl­phen­oxy)cyclo­tri­phos­phazene (2):**

The aryl­oxide was prepared by treatment of 4-tri­fluoro­methyl­phenol (1.63 g, 10 mmol) with a suspension of NaH [60% dispersion in mineral oil (0.39 g, 9.9 mmol)] in 50 ml of THF. To the stirred solution of 4-tri­fluoro­methyl­phenoxide was added a solution of hexa­chloro­cyclo­triphosphazene (0.50 g, 1.4 mmol) in 15 ml of THF and the mixture was stirred at room temperature overnight. The purification steps of this compound were identical to those of compound **1** to yield colorless cubes of **2**.

**Synthesis of hexa­kis­(3,5-bis-tri­fluoro­methyl­phen­oxy)cyclo­triphosphazene (3):**

A stirred suspension of NaH [60% dispersion in mineral oil (0.19 g, 4.9 mmol)] in 25 ml of THF was treated with liquid 3,5-bis-tri­fluoro­methyl­phenol (0.767 ml, 5.0 mmol) by dropwise addition. The resulting aryl­oxide solution was then added to a stirred solution of hexa­chloro­cyclo­triphosphazene (0.25 g, 0.72 mmol) in 25 ml of THF and the reaction mixture was stirred at room temperature overnight. The mixture was concentrated by rotary evaporation and the residue was dissolved in 40 ml di­chloro­methane. The di­chloro­methane solution was washed with 40 ml of deionized water, followed by 20 ml of 5% HCl, and finally rinsed with 40 ml of deionized water. The organic layer was dried over MgSO_4_ and the di­chloro­methane was removed by rotary evaporation to yield a colorless oil, which crystallized as colorless needles of **3** after standing for several hours. The crystals were rinsed with cold methanol and then dried under vacuum.

The NMR data for **1**–**3** are as follows: **1**: ^31^P 10.6 1 ppm (in chloro­form-*d*); ^19^F −153.52 (*d*, 2F), −157.53 (*t*, 1F), −161.79 (*t*, 2F); **2**: ^31^P 8.64 ppm (in chloro­form-*d*); ^1^H 747(*d*, 2H), 706 (d 2H); ^19^F −62.79 (*s*); **3**: ^31^P 7.71 ppm (in chloro­form-*d*); ^1^H 7.74 (*s* 1H), 7.52 (*s*, 2H); ^19^F −63.85 (*s*).

## Refinement   

Crystal data, data collection and structure refinement details for all three structures are summarized in Table 3 ▸. The hydrogen atoms in **2** and **3** were placed geometrically (C—H = 0.93 Å) and refined as riding on their parent atoms with *U*_iso_(H) = 1.2*U*_eq_(C).

## Supplementary Material

Crystal structure: contains datablock(s) 1, 2, 3. DOI: 10.1107/S2056989019012933/hb7853sup1.cif

Structure factors: contains datablock(s) 1. DOI: 10.1107/S2056989019012933/hb78531sup2.hkl

Supporting information file. DOI: 10.1107/S2056989019012933/hb78531sup5.mol

Structure factors: contains datablock(s) 2. DOI: 10.1107/S2056989019012933/hb78532sup4.hkl

Supporting information file. DOI: 10.1107/S2056989019012933/hb78532sup6.mol

Structure factors: contains datablock(s) 3. DOI: 10.1107/S2056989019012933/hb78533sup3.hkl

Supporting information file. DOI: 10.1107/S2056989019012933/hb78533sup7.mol

Additional supporting information:  crystallographic information; 3D view; checkCIF report

## Figures and Tables

**Figure 2:**
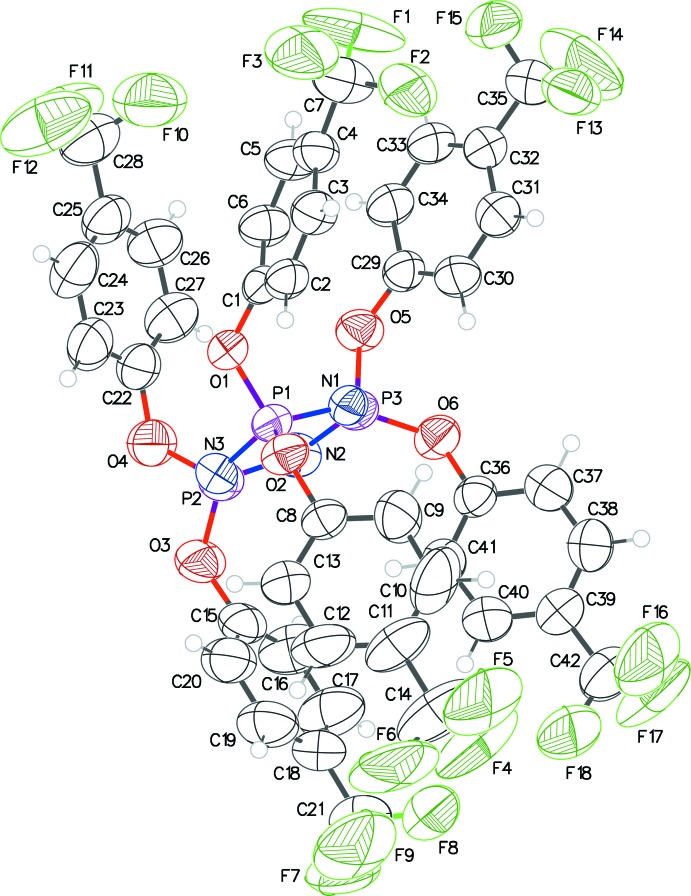
The mol­ecular structure of **2** with displacement ellipsoids drawn at the 50% probability level.

**Figure 3:**
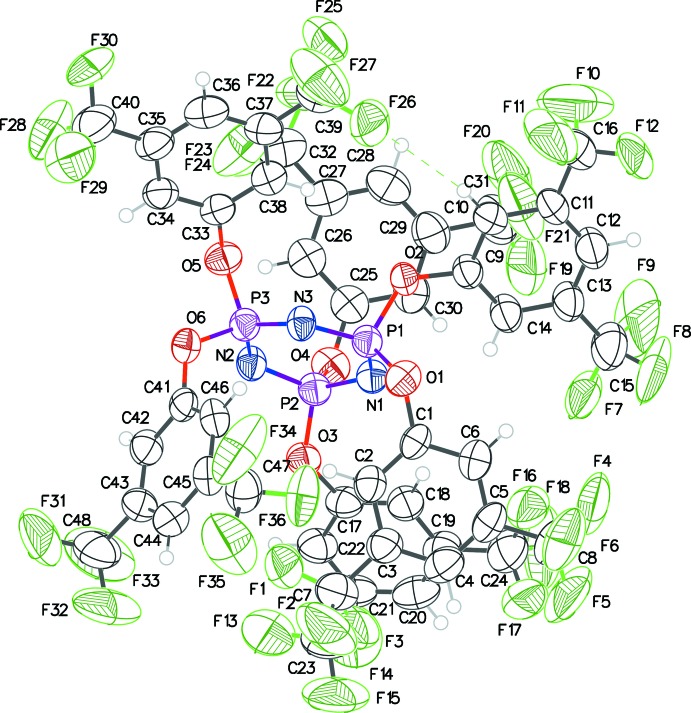
The mol­ecular structure of **3** with displacement ellipsoids drawn at the 50% probability level. The intra­molecular C—H⋯F inter­action is indicated by dashed lines.

**Table 1:** Hydrogen-bond geometry (Å, °) for (2)

*D*—H⋯*A*	*D*—H	H⋯*A*	*D*⋯*A*	*D*—H⋯*A*
C13—H13⋯F7*A* ^i^	0.93	2.64	3.475 (14)	149
C13—H13⋯F7*B* ^i^	0.93	2.52	3.316 (11)	144
C37—H37⋯F7*A* ^ii^	0.93	2.33	3.149 (8)	146

Symmetry codes: (i) 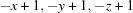; (ii) 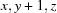.

**Table 2:** Hydrogen-bond geometry (Å, °) for (3)

*D*—H⋯*A*	*D*—H	H⋯*A*	*D*⋯*A*	*D*—H⋯*A*
C10—H10⋯F26	0.93	2.58	3.478 (10)	163
C30—H30⋯F6*A* ^i^	0.93	2.69	3.379 (14)	132
C36—H36⋯F32*A* ^ii^	0.93	2.59	3.40 (2)	146
C44—H44⋯F29^iii^	0.93	2.67	3.521 (13)	152

Symmetry codes: (i) 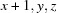; (ii) 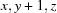; (iii) 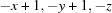.

**Table 3:** Experimental details

	(1)	(2)	(3)
Crystal data			
Chemical formula	C_36_F_30_N_3_O_6_P_3_	C_42_H_24_F_18_N_3_O_6_P_3_	C_48_H_18_F_36_N_3_O_6_P_3_
*M*_r_	1233.30	1101.55	1509.56
Crystal system, space group	Monoclinic, *P*2_1_/*c*	Triclinic, *P* 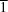	Triclinic, *P* 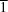
Temperature (K)	298	298	298
*a*, *b*, *c* (Å)	8.1111 (17), 44.054 (9), 12.259 (2)	13.4906 (11), 13.6573 (11), 14.9377 (13)	8.9782 (14), 13.947 (2), 23.205 (4)
α, β, γ (°)	90, 109.612 (12), 90	65.253 (2), 68.874 (2), 71.715 (2)	97.276 (6), 93.155 (6), 91.615 (6)
*V* (Å^3^)	4126.3 (14)	2288.0 (3)	2876.2 (8)
*Z*	4	2	2
Radiation type	Mo *K*α	Mo *K*α	Mo *K*α
μ (mm^−1^)	0.33	0.25	0.27
Crystal size (mm)	0.22 × 0.09 × 0.07	0.28 × 0.23 × 0.22	0.18 × 0.09 × 0.08

Data collection			
Diffractometer	Bruker SMART CCD area detector	Bruker SMART CCD area detector	Bruker SMART CCD area detector
Absorption correction	Multi-scan (*SADABS*; Bruker, 2001 ▸)	Multi-scan (*SADABS*; Bruker, 2001 ▸)	Multi-scan (*SADABS*; Bruker, 2001 ▸)
*T*_min_, *T*_max_	0.736, 0.9	0.727, 0.9	0.629, 0.9
No. of measured, independent and observed [*I* > 2σ(*I*)] reflections	35241, 10189, 6392	21657, 11015, 6744	28557, 14187, 6133
*R*_int_	0.048	0.019	0.046
(sin θ/λ)_max_ (Å^−1^)	0.667	0.666	0.675

Refinement			
*R*[*F* ^2^ > 2σ(*F* ^2^)], *wR*(*F* ^2^), *S*	0.065, 0.153, 1.04	0.062, 0.195, 0.98	0.063, 0.160, 0.95
No. of reflections	10189	11015	14187
No. of parameters	703	817	1201
No. of restraints	0	462	813
H-atom treatment	–	H-atom parameters constrained	H-atom parameters constrained
Δρ_max_, Δρ_min_ (e Å^−3^)	0.37, −0.28	0.32, −0.27	0.28, −0.32

Computer programs: *SMART* and *SAINT* (Bruker, 2001 ▸), *SHELXS97* (Sheldrick, 2008 ▸), *SHELXL* (Sheldrick, 2015 ▸) and *OLEX2* (Dolomanov *et al.*, 2009 ▸).

## supplementary crystallographic information

### Hexakis(pentafluorophenoxy)cyclotriphosphazene (1) Crystal data

**Table:**

C_36_F_30_N_3_O_6_P_3_	*F*(000) = 2400
*M**_r_* = 1233.30	*D*_x_ = 1.985 Mg m^−^^3^
Monoclinic, *P*2_1_/*c*	Mo *K*α radiation, λ = 0.71073 Å
*a* = 8.1111 (17) Å	Cell parameters from 4693 reflections
*b* = 44.054 (9) Å	θ = 2.7–27.8°
*c* = 12.259 (2) Å	µ = 0.33 mm^−^^1^
β = 109.612 (12)°	*T* = 298 K
*V* = 4126.3 (14) Å^3^	Block, colorless
*Z* = 4	0.22 × 0.09 × 0.07 mm

### Hexakis(pentafluorophenoxy)cyclotriphosphazene (1) Data collection

**Table:**

Bruker SMART CCD area detector diffractometer	10189 independent reflections
Radiation source: fine-focus sealed tube	6392 reflections with *I* > 2σ(*I*)
Graphite monochromator	*R*_int_ = 0.048
phi and ω scans	θ_max_ = 28.3°, θ_min_ = 1.8°
Absorption correction: multi-scan (SADABS; Bruker, 2001)	*h* = −10→10
*T*_min_ = 0.736, *T*_max_ = 0.9	*k* = −58→50
35241 measured reflections	*l* = −16→16

### Hexakis(pentafluorophenoxy)cyclotriphosphazene (1) Refinement

**Table:**

Refinement on *F*^2^	703 parameters
Least-squares matrix: full	0 restraints
*R*[*F*^2^ > 2σ(*F*^2^)] = 0.065	*w* = 1/[σ^2^(*F*_o_^2^) + (0.0615*P*)^2^ + 1.7223*P*] where *P* = (*F*_o_^2^ + 2*F*_c_^2^)/3
*wR*(*F*^2^) = 0.153	(Δ/σ)_max_ < 0.001
*S* = 1.04	Δρ_max_ = 0.37 e Å^−^^3^
10189 reflections	Δρ_min_ = −0.28 e Å^−^^3^

### Hexakis(pentafluorophenoxy)cyclotriphosphazene (1) Special details

**Table:**

Geometry. All esds (except the esd in the dihedral angle between two l.s. planes) are estimated using the full covariance matrix. The cell esds are taken into account individually in the estimation of esds in distances, angles and torsion angles; correlations between esds in cell parameters are only used when they are defined by crystal symmetry. An approximate (isotropic) treatment of cell esds is used for estimating esds involving l.s. planes.

### Hexakis(pentafluorophenoxy)cyclotriphosphazene (1) Fractional atomic coordinates and isotropic or equivalent isotropic displacement parameters (Å^2^)

**Table:**

	*x*	*y*	*z*	*U*_iso_*/*U*_eq_	
C1	0.6926 (4)	0.09424 (7)	1.1812 (3)	0.0416 (7)	
C2	0.5803 (4)	0.07350 (7)	1.2016 (3)	0.0470 (8)	
C3	0.5875 (5)	0.06715 (8)	1.3133 (3)	0.0554 (9)	
C4	0.7097 (6)	0.08083 (9)	1.4052 (3)	0.0610 (10)	
C5	0.8280 (5)	0.10073 (9)	1.3867 (3)	0.0573 (9)	
C6	0.8207 (4)	0.10717 (8)	1.2747 (3)	0.0475 (8)	
C7	0.5605 (4)	0.17945 (7)	1.0959 (3)	0.0460 (8)	
C8	0.5722 (5)	0.18524 (8)	1.2088 (3)	0.0497 (8)	
C9	0.4951 (5)	0.21051 (8)	1.2361 (3)	0.0582 (10)	
C10	0.4057 (6)	0.23023 (9)	1.1506 (4)	0.0702 (11)	
C11	0.3920 (6)	0.22461 (9)	1.0372 (4)	0.0736 (12)	
C12	0.4707 (6)	0.19951 (9)	1.0112 (3)	0.0621 (10)	
C13	0.3912 (4)	0.07382 (8)	0.6439 (3)	0.0466 (8)	
C14	0.4062 (5)	0.05131 (8)	0.7251 (3)	0.0494 (8)	
C15	0.3329 (5)	0.02319 (9)	0.6940 (3)	0.0569 (9)	
C16	0.2437 (5)	0.01726 (9)	0.5794 (4)	0.0641 (10)	
C17	0.2247 (5)	0.03944 (9)	0.4968 (3)	0.0618 (10)	
C18	0.2987 (5)	0.06757 (8)	0.5299 (3)	0.0544 (9)	
C19	0.3286 (4)	0.16421 (8)	0.5883 (3)	0.0477 (8)	
C20	0.3597 (5)	0.15734 (9)	0.4861 (3)	0.0546 (9)	
C21	0.2459 (6)	0.16755 (11)	0.3823 (3)	0.0688 (12)	
C22	0.1052 (6)	0.18442 (11)	0.3783 (4)	0.0770 (14)	
C23	0.0725 (5)	0.19159 (9)	0.4787 (4)	0.0701 (12)	
C24	0.1862 (5)	0.18160 (8)	0.5842 (3)	0.0549 (9)	
C25	0.0555 (4)	0.06138 (7)	0.9107 (3)	0.0397 (7)	
C26	0.0353 (4)	0.06203 (8)	1.0179 (3)	0.0484 (8)	
C27	−0.0802 (5)	0.04288 (9)	1.0433 (3)	0.0568 (10)	
C28	−0.1764 (5)	0.02322 (9)	0.9628 (4)	0.0620 (10)	
C29	−0.1628 (5)	0.02253 (8)	0.8558 (4)	0.0613 (10)	
C30	−0.0443 (4)	0.04154 (8)	0.8297 (3)	0.0506 (8)	
C31	−0.0249 (4)	0.15380 (7)	0.8629 (3)	0.0408 (7)	
C32	−0.1235 (4)	0.16325 (9)	0.7536 (3)	0.0540 (9)	
C33	−0.1967 (5)	0.19206 (10)	0.7379 (4)	0.0681 (11)	
C34	−0.1686 (6)	0.21080 (9)	0.8302 (5)	0.0776 (13)	
C35	−0.0736 (6)	0.20128 (11)	0.9383 (4)	0.0848 (14)	
C36	−0.0014 (5)	0.17292 (9)	0.9550 (3)	0.0629 (10)	
F1	0.4652 (3)	0.05897 (5)	1.11273 (18)	0.0638 (6)	
F2	0.4764 (3)	0.04710 (5)	1.3322 (2)	0.0827 (7)	
F3	0.7157 (4)	0.07480 (6)	1.51341 (19)	0.0915 (8)	
F4	0.9493 (3)	0.11414 (6)	1.47566 (18)	0.0846 (7)	
F5	0.9380 (3)	0.12597 (5)	1.25684 (17)	0.0677 (6)	
F6	0.6578 (3)	0.16612 (5)	1.29261 (17)	0.0672 (6)	
F7	0.5068 (4)	0.21555 (5)	1.3460 (2)	0.0864 (8)	
F8	0.3293 (4)	0.25475 (6)	1.1773 (3)	0.1067 (9)	
F9	0.3015 (5)	0.24383 (6)	0.9530 (3)	0.1180 (11)	
F10	0.4592 (4)	0.19441 (6)	0.90053 (19)	0.0906 (8)	
F11	0.4950 (3)	0.05731 (5)	0.83754 (17)	0.0646 (6)	
F12	0.3469 (3)	0.00219 (5)	0.7744 (2)	0.0839 (7)	
F13	0.1757 (3)	−0.01046 (6)	0.5482 (2)	0.0913 (8)	
F14	0.1356 (4)	0.03367 (6)	0.3857 (2)	0.0911 (8)	
F15	0.2806 (3)	0.08866 (5)	0.44835 (17)	0.0727 (6)	
F16	0.5005 (3)	0.14165 (6)	0.48793 (17)	0.0741 (6)	
F17	0.2745 (4)	0.16014 (7)	0.28379 (18)	0.1002 (9)	
F18	−0.0045 (4)	0.19480 (7)	0.2765 (2)	0.1156 (11)	
F19	−0.0640 (4)	0.20851 (7)	0.4760 (3)	0.1098 (10)	
F20	0.1589 (3)	0.18879 (5)	0.6823 (2)	0.0692 (6)	
F21	0.1291 (3)	0.08154 (6)	1.09856 (19)	0.0790 (7)	
F22	−0.0943 (4)	0.04346 (6)	1.1492 (2)	0.0922 (8)	
F23	−0.2883 (4)	0.00434 (6)	0.9892 (3)	0.1051 (10)	
F24	−0.2579 (3)	0.00299 (7)	0.7757 (3)	0.1065 (10)	
F25	−0.0271 (3)	0.03995 (6)	0.72509 (18)	0.0796 (7)	
F26	−0.1516 (3)	0.14481 (7)	0.66480 (19)	0.0926 (8)	
F27	−0.2941 (4)	0.20106 (7)	0.6316 (2)	0.1131 (10)	
F28	−0.2407 (5)	0.23864 (7)	0.8134 (3)	0.1388 (14)	
F29	−0.0517 (5)	0.21937 (8)	1.0304 (3)	0.1567 (17)	
F30	0.0883 (4)	0.16350 (7)	1.0612 (2)	0.1096 (11)	
N1	0.5949 (3)	0.12681 (6)	0.8763 (2)	0.0421 (6)	
N2	0.2484 (3)	0.11962 (6)	0.7635 (2)	0.0423 (6)	
N3	0.3687 (3)	0.12301 (6)	0.9952 (2)	0.0415 (6)	
O1	0.6882 (3)	0.10044 (5)	1.06885 (17)	0.0434 (5)	
O2	0.6489 (3)	0.15494 (5)	1.07273 (18)	0.0477 (5)	
O3	0.4738 (3)	0.10159 (5)	0.67212 (18)	0.0501 (6)	
O4	0.4493 (3)	0.15599 (5)	0.69450 (17)	0.0477 (6)	
O5	0.1827 (3)	0.07727 (5)	0.8847 (2)	0.0520 (6)	
O6	0.0334 (3)	0.12420 (5)	0.8812 (2)	0.0483 (6)	
P1	0.56188 (10)	0.12633 (2)	0.99536 (6)	0.03754 (19)	
P2	0.43660 (11)	0.12538 (2)	0.75940 (6)	0.0398 (2)	
P3	0.21764 (10)	0.11254 (2)	0.88150 (7)	0.0386 (2)	

### Hexakis(pentafluorophenoxy)cyclotriphosphazene (1) Atomic displacement parameters (Å^2^)

**Table:**

	*U*^11^	*U*^22^	*U*^33^	*U*^12^	*U*^13^	*U*^23^
C1	0.0455 (18)	0.0390 (18)	0.0401 (16)	0.0065 (15)	0.0142 (14)	0.0016 (13)
C2	0.0475 (19)	0.0378 (18)	0.0529 (19)	0.0032 (15)	0.0133 (15)	0.0001 (15)
C3	0.066 (2)	0.040 (2)	0.065 (2)	0.0042 (18)	0.029 (2)	0.0117 (17)
C4	0.085 (3)	0.061 (2)	0.0419 (19)	0.017 (2)	0.0273 (19)	0.0082 (17)
C5	0.063 (2)	0.062 (2)	0.0373 (18)	0.005 (2)	0.0039 (16)	−0.0046 (16)
C6	0.047 (2)	0.046 (2)	0.0454 (18)	0.0015 (16)	0.0109 (15)	0.0022 (15)
C7	0.0481 (19)	0.0377 (19)	0.0521 (19)	−0.0083 (15)	0.0165 (15)	−0.0020 (15)
C8	0.054 (2)	0.040 (2)	0.0547 (19)	−0.0068 (16)	0.0190 (17)	−0.0011 (16)
C9	0.075 (3)	0.046 (2)	0.065 (2)	−0.0116 (19)	0.038 (2)	−0.0116 (18)
C10	0.091 (3)	0.037 (2)	0.094 (3)	0.005 (2)	0.046 (3)	−0.006 (2)
C11	0.101 (3)	0.042 (2)	0.079 (3)	0.011 (2)	0.031 (3)	0.016 (2)
C12	0.086 (3)	0.046 (2)	0.054 (2)	0.005 (2)	0.023 (2)	0.0002 (17)
C13	0.050 (2)	0.048 (2)	0.0482 (18)	0.0033 (16)	0.0245 (15)	−0.0031 (15)
C14	0.051 (2)	0.056 (2)	0.0454 (18)	0.0130 (17)	0.0211 (15)	0.0036 (16)
C15	0.062 (2)	0.049 (2)	0.065 (2)	0.0109 (18)	0.0296 (19)	0.0074 (18)
C16	0.062 (2)	0.049 (2)	0.087 (3)	−0.0019 (19)	0.033 (2)	−0.009 (2)
C17	0.063 (2)	0.068 (3)	0.056 (2)	−0.005 (2)	0.0223 (19)	−0.0138 (19)
C18	0.067 (2)	0.056 (2)	0.0445 (19)	0.0048 (19)	0.0246 (17)	0.0027 (16)
C19	0.0469 (19)	0.049 (2)	0.0431 (17)	−0.0138 (16)	0.0097 (15)	0.0085 (15)
C20	0.052 (2)	0.065 (2)	0.0438 (18)	−0.0195 (19)	0.0121 (16)	0.0061 (16)
C21	0.066 (3)	0.089 (3)	0.046 (2)	−0.033 (2)	0.0110 (19)	0.014 (2)
C22	0.073 (3)	0.076 (3)	0.060 (3)	−0.023 (2)	−0.006 (2)	0.033 (2)
C23	0.057 (3)	0.051 (2)	0.090 (3)	−0.001 (2)	0.009 (2)	0.018 (2)
C24	0.059 (2)	0.044 (2)	0.062 (2)	−0.0089 (18)	0.0194 (18)	0.0084 (17)
C25	0.0337 (16)	0.0350 (17)	0.0515 (18)	0.0007 (13)	0.0157 (14)	0.0020 (14)
C26	0.049 (2)	0.047 (2)	0.0505 (19)	0.0088 (16)	0.0181 (16)	0.0007 (15)
C27	0.063 (2)	0.056 (2)	0.061 (2)	0.0210 (19)	0.0337 (19)	0.0187 (18)
C28	0.054 (2)	0.043 (2)	0.103 (3)	0.0072 (18)	0.045 (2)	0.015 (2)
C29	0.047 (2)	0.043 (2)	0.091 (3)	−0.0061 (17)	0.019 (2)	−0.016 (2)
C30	0.0438 (19)	0.057 (2)	0.0517 (19)	0.0038 (17)	0.0164 (16)	−0.0073 (16)
C31	0.0313 (16)	0.0438 (19)	0.0482 (17)	0.0016 (14)	0.0145 (13)	0.0014 (14)
C32	0.0404 (19)	0.064 (2)	0.052 (2)	0.0008 (17)	0.0086 (16)	−0.0051 (17)
C33	0.046 (2)	0.076 (3)	0.072 (3)	0.011 (2)	0.0061 (19)	0.024 (2)
C34	0.071 (3)	0.044 (2)	0.108 (4)	0.016 (2)	0.018 (3)	0.004 (2)
C35	0.085 (3)	0.071 (3)	0.087 (3)	0.022 (3)	0.014 (3)	−0.027 (3)
C36	0.060 (2)	0.068 (3)	0.054 (2)	0.013 (2)	0.0106 (18)	−0.0071 (19)
F1	0.0627 (13)	0.0517 (13)	0.0676 (13)	−0.0130 (10)	0.0094 (11)	−0.0062 (10)
F2	0.0933 (18)	0.0708 (16)	0.0991 (18)	−0.0122 (13)	0.0523 (15)	0.0165 (13)
F3	0.130 (2)	0.0991 (19)	0.0522 (13)	0.0106 (17)	0.0400 (14)	0.0150 (12)
F4	0.0969 (18)	0.0943 (18)	0.0446 (12)	−0.0045 (15)	−0.0001 (12)	−0.0116 (12)
F5	0.0548 (13)	0.0764 (15)	0.0632 (13)	−0.0181 (11)	0.0081 (10)	0.0023 (11)
F6	0.0811 (15)	0.0679 (14)	0.0505 (11)	0.0101 (12)	0.0195 (11)	0.0037 (10)
F7	0.124 (2)	0.0742 (17)	0.0773 (15)	−0.0031 (15)	0.0556 (15)	−0.0204 (12)
F8	0.145 (3)	0.0604 (16)	0.131 (2)	0.0308 (17)	0.068 (2)	−0.0052 (15)
F9	0.167 (3)	0.0779 (19)	0.108 (2)	0.0553 (19)	0.045 (2)	0.0362 (16)
F10	0.138 (2)	0.0800 (17)	0.0510 (13)	0.0280 (16)	0.0274 (14)	0.0122 (11)
F11	0.0714 (14)	0.0710 (14)	0.0505 (12)	0.0135 (11)	0.0194 (10)	0.0088 (10)
F12	0.1004 (19)	0.0582 (15)	0.0973 (18)	0.0091 (13)	0.0389 (15)	0.0250 (13)
F13	0.0918 (19)	0.0572 (16)	0.117 (2)	−0.0105 (13)	0.0252 (16)	−0.0142 (14)
F14	0.108 (2)	0.0894 (19)	0.0641 (15)	−0.0214 (15)	0.0137 (14)	−0.0236 (13)
F15	0.0969 (17)	0.0714 (15)	0.0477 (12)	−0.0055 (13)	0.0213 (11)	0.0043 (10)
F16	0.0680 (15)	0.1052 (19)	0.0565 (12)	−0.0079 (14)	0.0309 (11)	0.0023 (12)
F17	0.0997 (19)	0.155 (3)	0.0414 (12)	−0.0437 (18)	0.0177 (12)	0.0091 (14)
F18	0.100 (2)	0.131 (3)	0.0808 (17)	−0.0171 (18)	−0.0158 (15)	0.0543 (17)
F19	0.084 (2)	0.089 (2)	0.138 (3)	0.0258 (16)	0.0137 (18)	0.0305 (18)
F20	0.0739 (15)	0.0547 (13)	0.0833 (15)	0.0004 (11)	0.0322 (12)	−0.0005 (11)
F21	0.0782 (16)	0.0930 (18)	0.0682 (14)	−0.0034 (13)	0.0276 (12)	−0.0285 (13)
F22	0.108 (2)	0.109 (2)	0.0837 (16)	0.0342 (16)	0.0637 (15)	0.0331 (14)
F23	0.0888 (19)	0.0729 (17)	0.179 (3)	−0.0060 (14)	0.078 (2)	0.0311 (18)
F24	0.0753 (17)	0.101 (2)	0.142 (2)	−0.0374 (16)	0.0350 (17)	−0.0549 (19)
F25	0.0721 (15)	0.109 (2)	0.0579 (13)	−0.0093 (14)	0.0224 (11)	−0.0215 (12)
F26	0.0747 (16)	0.120 (2)	0.0586 (13)	0.0176 (15)	−0.0094 (12)	−0.0294 (14)
F27	0.092 (2)	0.131 (3)	0.0925 (19)	0.0332 (18)	−0.0006 (15)	0.0503 (18)
F28	0.125 (3)	0.0636 (19)	0.202 (4)	0.0419 (18)	0.021 (2)	0.009 (2)
F29	0.185 (4)	0.124 (3)	0.129 (3)	0.061 (3)	0.010 (2)	−0.068 (2)
F30	0.129 (2)	0.141 (3)	0.0469 (13)	0.062 (2)	0.0131 (14)	−0.0107 (14)
N1	0.0348 (14)	0.0533 (17)	0.0400 (13)	−0.0002 (12)	0.0150 (11)	0.0015 (12)
N2	0.0359 (14)	0.0434 (16)	0.0436 (14)	−0.0005 (12)	0.0084 (11)	0.0010 (11)
N3	0.0432 (14)	0.0477 (16)	0.0378 (13)	0.0003 (12)	0.0191 (11)	0.0023 (11)
O1	0.0445 (12)	0.0474 (13)	0.0381 (11)	0.0057 (10)	0.0135 (9)	−0.0002 (9)
O2	0.0435 (13)	0.0471 (14)	0.0503 (13)	−0.0012 (11)	0.0128 (10)	−0.0058 (10)
O3	0.0574 (15)	0.0533 (15)	0.0475 (13)	−0.0046 (12)	0.0280 (11)	−0.0041 (10)
O4	0.0497 (13)	0.0525 (14)	0.0394 (11)	−0.0095 (11)	0.0130 (10)	0.0041 (10)
O5	0.0466 (13)	0.0388 (13)	0.0809 (16)	−0.0011 (10)	0.0352 (12)	0.0006 (11)
O6	0.0366 (12)	0.0435 (13)	0.0700 (15)	0.0032 (10)	0.0246 (11)	0.0038 (11)
P1	0.0361 (4)	0.0414 (5)	0.0356 (4)	0.0004 (4)	0.0126 (3)	−0.0002 (3)
P2	0.0392 (4)	0.0463 (5)	0.0348 (4)	−0.0025 (4)	0.0137 (3)	0.0017 (3)
P3	0.0342 (4)	0.0372 (5)	0.0473 (4)	0.0003 (3)	0.0174 (3)	0.0012 (3)

### Hexakis(pentafluorophenoxy)cyclotriphosphazene (1) Geometric parameters (Å, º)

**Table:**

C1—C2	1.371 (5)	C21—F17	1.344 (5)
C1—C6	1.385 (4)	C22—C23	1.380 (6)
C1—O1	1.392 (3)	C22—F18	1.346 (4)
C2—C3	1.379 (5)	C23—C24	1.385 (5)
C2—F1	1.336 (4)	C23—F19	1.326 (5)
C3—C4	1.366 (5)	C24—F20	1.331 (4)
C3—F2	1.336 (4)	C25—C26	1.379 (4)
C4—C5	1.374 (6)	C25—C30	1.365 (4)
C4—F3	1.337 (4)	C25—O5	1.370 (4)
C5—C6	1.383 (5)	C26—C27	1.372 (5)
C5—F4	1.337 (4)	C26—F21	1.338 (4)
C6—F5	1.334 (4)	C27—C28	1.347 (6)
C7—C8	1.379 (4)	C27—F22	1.342 (4)
C7—C12	1.372 (5)	C28—C29	1.354 (6)
C7—O2	1.378 (4)	C28—F23	1.348 (4)
C8—C9	1.372 (5)	C29—C30	1.390 (5)
C8—F6	1.330 (4)	C29—F24	1.338 (4)
C9—C10	1.367 (6)	C30—F25	1.338 (4)
C9—F7	1.337 (4)	C31—C32	1.374 (4)
C10—C11	1.380 (6)	C31—C36	1.369 (5)
C10—F8	1.340 (4)	C31—O6	1.380 (4)
C11—C12	1.367 (5)	C32—C33	1.387 (5)
C11—F9	1.346 (5)	C32—F26	1.315 (4)
C12—F10	1.347 (4)	C33—C34	1.358 (6)
C13—C14	1.381 (5)	C33—F27	1.337 (4)
C13—C18	1.374 (5)	C34—C35	1.357 (6)
C13—O3	1.382 (4)	C34—F28	1.344 (5)
C14—C15	1.372 (5)	C35—C36	1.366 (6)
C14—F11	1.350 (4)	C35—F29	1.343 (5)
C15—C16	1.372 (5)	C36—F30	1.327 (4)
C15—F12	1.328 (4)	N1—P1	1.570 (2)
C16—C17	1.378 (6)	N1—P2	1.572 (3)
C16—F13	1.342 (4)	N2—P2	1.565 (3)
C17—C18	1.377 (5)	N2—P3	1.579 (3)
C17—F14	1.334 (4)	N3—P1	1.573 (3)
C18—F15	1.336 (4)	N3—P3	1.584 (3)
C19—C20	1.391 (5)	O1—P1	1.594 (2)
C19—C24	1.373 (5)	O2—P1	1.593 (2)
C19—O4	1.389 (4)	O3—P2	1.597 (2)
C20—C21	1.373 (5)	O4—P2	1.586 (2)
C20—F16	1.328 (4)	O5—P3	1.582 (2)
C21—C22	1.349 (6)	O6—P3	1.579 (2)
			
C2—C1—C6	118.8 (3)	F19—C23—C24	119.4 (4)
C2—C1—O1	120.9 (3)	C19—C24—C23	119.9 (4)
C6—C1—O1	120.1 (3)	F20—C24—C19	119.6 (3)
C1—C2—C3	120.5 (3)	F20—C24—C23	120.5 (4)
F1—C2—C1	119.7 (3)	C30—C25—C26	118.3 (3)
F1—C2—C3	119.8 (3)	C30—C25—O5	117.9 (3)
C4—C3—C2	120.5 (3)	O5—C25—C26	123.4 (3)
F2—C3—C2	120.0 (3)	C27—C26—C25	120.8 (3)
F2—C3—C4	119.6 (3)	F21—C26—C25	119.8 (3)
C3—C4—C5	119.9 (3)	F21—C26—C27	119.4 (3)
F3—C4—C3	120.4 (4)	C28—C27—C26	120.1 (3)
F3—C4—C5	119.8 (4)	F22—C27—C26	119.6 (4)
C4—C5—C6	119.7 (3)	F22—C27—C28	120.3 (4)
F4—C5—C4	120.7 (3)	C27—C28—C29	120.6 (3)
F4—C5—C6	119.6 (4)	C27—C28—F23	119.5 (4)
C5—C6—C1	120.5 (3)	F23—C28—C29	119.9 (4)
F5—C6—C1	119.9 (3)	C28—C29—C30	119.8 (3)
F5—C6—C5	119.7 (3)	F24—C29—C28	120.7 (4)
C12—C7—C8	118.8 (3)	F24—C29—C30	119.5 (4)
C12—C7—O2	122.0 (3)	C25—C30—C29	120.4 (3)
O2—C7—C8	119.1 (3)	F25—C30—C25	120.2 (3)
C9—C8—C7	120.7 (3)	F25—C30—C29	119.4 (3)
F6—C8—C7	120.0 (3)	C32—C31—O6	120.0 (3)
F6—C8—C9	119.2 (3)	C36—C31—C32	119.5 (3)
C10—C9—C8	119.8 (3)	C36—C31—O6	120.1 (3)
F7—C9—C8	119.8 (4)	C31—C32—C33	119.6 (3)
F7—C9—C10	120.5 (4)	F26—C32—C31	120.2 (3)
C9—C10—C11	120.1 (4)	F26—C32—C33	120.2 (3)
F8—C10—C9	119.7 (4)	C34—C33—C32	119.8 (4)
F8—C10—C11	120.3 (4)	F27—C33—C32	119.5 (4)
C12—C11—C10	119.6 (4)	F27—C33—C34	120.7 (4)
F9—C11—C10	119.9 (4)	C35—C34—C33	120.5 (4)
F9—C11—C12	120.5 (4)	F28—C34—C33	119.0 (4)
C11—C12—C7	121.0 (3)	F28—C34—C35	120.5 (4)
F10—C12—C7	119.7 (3)	C34—C35—C36	120.2 (4)
F10—C12—C11	119.3 (3)	F29—C35—C34	120.6 (4)
C14—C13—O3	122.5 (3)	F29—C35—C36	119.2 (4)
C18—C13—C14	118.4 (3)	C35—C36—C31	120.4 (4)
C18—C13—O3	119.0 (3)	F30—C36—C31	119.7 (3)
C15—C14—C13	121.5 (3)	F30—C36—C35	119.9 (4)
F11—C14—C13	118.9 (3)	P1—N1—P2	120.34 (16)
F11—C14—C15	119.6 (3)	P2—N2—P3	121.16 (16)
C14—C15—C16	119.1 (3)	P1—N3—P3	120.25 (15)
F12—C15—C14	120.1 (3)	C1—O1—P1	119.83 (18)
F12—C15—C16	120.8 (4)	C7—O2—P1	125.9 (2)
C15—C16—C17	120.5 (4)	C13—O3—P2	124.36 (19)
F13—C16—C15	119.3 (4)	C19—O4—P2	123.23 (19)
F13—C16—C17	120.1 (4)	C25—O5—P3	131.5 (2)
C18—C17—C16	119.4 (3)	C31—O6—P3	125.49 (19)
F14—C17—C16	120.2 (4)	N1—P1—N3	118.74 (13)
F14—C17—C18	120.3 (4)	N1—P1—O1	104.91 (13)
C13—C18—C17	121.0 (3)	N1—P1—O2	111.30 (13)
F15—C18—C13	120.5 (3)	N3—P1—O1	112.64 (13)
F15—C18—C17	118.5 (3)	N3—P1—O2	108.43 (13)
C24—C19—C20	119.6 (3)	O2—P1—O1	99.06 (12)
C24—C19—O4	119.9 (3)	N1—P2—O3	110.92 (14)
O4—C19—C20	120.2 (3)	N1—P2—O4	104.64 (13)
C21—C20—C19	119.6 (4)	N2—P2—N1	118.94 (13)
F16—C20—C19	120.8 (3)	N2—P2—O3	108.71 (13)
F16—C20—C21	119.5 (3)	N2—P2—O4	112.47 (13)
C22—C21—C20	120.8 (4)	O4—P2—O3	99.40 (12)
F17—C21—C20	119.2 (4)	N2—P3—N3	115.79 (13)
F17—C21—C22	120.0 (4)	N2—P3—O5	107.53 (13)
C21—C22—C23	120.4 (4)	N2—P3—O6	111.94 (13)
F18—C22—C21	120.6 (5)	O5—P3—N3	110.87 (14)
F18—C22—C23	119.0 (5)	O6—P3—N3	111.06 (13)
C22—C23—C24	119.6 (4)	O6—P3—O5	98.18 (12)
F19—C23—C22	121.0 (4)		
			
C1—C2—C3—C4	−1.7 (5)	C33—C34—C35—F29	−177.3 (5)
C1—C2—C3—F2	179.4 (3)	C34—C35—C36—C31	−0.3 (7)
C1—O1—P1—N1	179.4 (2)	C34—C35—C36—F30	−178.6 (4)
C1—O1—P1—N3	−50.1 (3)	C36—C31—C32—C33	0.5 (5)
C1—O1—P1—O2	64.3 (2)	C36—C31—C32—F26	−177.9 (3)
C2—C1—C6—C5	−4.1 (5)	C36—C31—O6—P3	−93.0 (4)
C2—C1—C6—F5	175.9 (3)	F1—C2—C3—C4	177.1 (3)
C2—C1—O1—P1	88.8 (3)	F1—C2—C3—F2	−1.8 (5)
C2—C3—C4—C5	−0.9 (6)	F2—C3—C4—C5	178.0 (3)
C2—C3—C4—F3	179.5 (3)	F2—C3—C4—F3	−1.6 (6)
C3—C4—C5—C6	1.0 (6)	F3—C4—C5—C6	−179.4 (3)
C3—C4—C5—F4	−179.5 (3)	F3—C4—C5—F4	0.1 (6)
C4—C5—C6—C1	1.5 (5)	F4—C5—C6—C1	−178.0 (3)
C4—C5—C6—F5	−178.5 (3)	F4—C5—C6—F5	2.0 (5)
C6—C1—C2—C3	4.2 (5)	F6—C8—C9—C10	−179.6 (3)
C6—C1—C2—F1	−174.6 (3)	F6—C8—C9—F7	−0.1 (5)
C6—C1—O1—P1	−96.5 (3)	F7—C9—C10—C11	−179.0 (4)
C7—C8—C9—C10	−0.1 (6)	F7—C9—C10—F8	0.0 (6)
C7—C8—C9—F7	179.4 (3)	F8—C10—C11—C12	179.9 (4)
C7—O2—P1—N1	107.8 (3)	F8—C10—C11—F9	0.0 (7)
C7—O2—P1—N3	−24.5 (3)	F9—C11—C12—C7	−178.7 (4)
C7—O2—P1—O1	−142.2 (2)	F9—C11—C12—F10	1.0 (7)
C8—C7—C12—C11	−0.9 (6)	F11—C14—C15—C16	−179.7 (3)
C8—C7—C12—F10	179.4 (3)	F11—C14—C15—F12	1.1 (5)
C8—C7—O2—P1	117.5 (3)	F12—C15—C16—C17	177.9 (4)
C8—C9—C10—C11	0.5 (6)	F12—C15—C16—F13	−2.5 (6)
C8—C9—C10—F8	179.5 (4)	F13—C16—C17—C18	−178.5 (3)
C9—C10—C11—C12	−1.1 (7)	F13—C16—C17—F14	1.4 (6)
C9—C10—C11—F9	178.9 (4)	F14—C17—C18—C13	−179.8 (3)
C10—C11—C12—C7	1.3 (7)	F14—C17—C18—F15	−0.7 (6)
C10—C11—C12—F10	−179.0 (4)	F16—C20—C21—C22	−177.7 (4)
C12—C7—C8—C9	0.3 (5)	F16—C20—C21—F17	3.3 (5)
C12—C7—C8—F6	179.7 (3)	F17—C21—C22—C23	178.6 (4)
C12—C7—O2—P1	−67.1 (4)	F17—C21—C22—F18	−2.2 (6)
C13—C14—C15—C16	0.5 (5)	F18—C22—C23—C24	−178.6 (3)
C13—C14—C15—F12	−178.8 (3)	F18—C22—C23—F19	−0.2 (6)
C13—O3—P2—N1	108.2 (3)	F19—C23—C24—C19	−179.6 (3)
C13—O3—P2—N2	−24.3 (3)	F19—C23—C24—F20	0.2 (5)
C13—O3—P2—O4	−142.1 (2)	F21—C26—C27—C28	179.6 (3)
C14—C13—C18—C17	−1.0 (5)	F21—C26—C27—F22	−1.6 (5)
C14—C13—C18—F15	179.9 (3)	F22—C27—C28—C29	180.0 (3)
C14—C13—O3—P2	−62.3 (4)	F22—C27—C28—F23	0.7 (5)
C14—C15—C16—C17	−1.3 (6)	F23—C28—C29—C30	−178.5 (3)
C14—C15—C16—F13	178.2 (3)	F23—C28—C29—F24	−0.8 (6)
C15—C16—C17—C18	1.1 (6)	F24—C29—C30—C25	−179.2 (3)
C15—C16—C17—F14	−179.0 (3)	F24—C29—C30—F25	−0.1 (5)
C16—C17—C18—C13	0.1 (6)	F26—C32—C33—C34	179.2 (4)
C16—C17—C18—F15	179.2 (3)	F26—C32—C33—F27	−0.8 (6)
C18—C13—C14—C15	0.7 (5)	F27—C33—C34—C35	178.2 (4)
C18—C13—C14—F11	−179.2 (3)	F27—C33—C34—F28	0.1 (7)
C18—C13—O3—P2	121.5 (3)	F28—C34—C35—C36	179.7 (4)
C19—C20—C21—C22	0.7 (6)	F28—C34—C35—F29	0.8 (8)
C19—C20—C21—F17	−178.3 (3)	F29—C35—C36—C31	178.6 (4)
C19—O4—P2—N1	−177.7 (2)	F29—C35—C36—F30	0.3 (7)
C19—O4—P2—N2	−47.3 (3)	O1—C1—C2—C3	178.9 (3)
C19—O4—P2—O3	67.6 (3)	O1—C1—C2—F1	0.1 (5)
C20—C19—C24—C23	1.5 (5)	O1—C1—C6—C5	−178.9 (3)
C20—C19—C24—F20	−178.4 (3)	O1—C1—C6—F5	1.1 (5)
C20—C19—O4—P2	−94.2 (3)	O2—C7—C8—C9	175.9 (3)
C20—C21—C22—C23	−0.4 (6)	O2—C7—C8—F6	−4.7 (5)
C20—C21—C22—F18	178.9 (3)	O2—C7—C12—C11	−176.4 (4)
C21—C22—C23—C24	0.6 (6)	O2—C7—C12—F10	3.9 (6)
C21—C22—C23—F19	179.0 (4)	O3—C13—C14—C15	−175.6 (3)
C22—C23—C24—C19	−1.2 (6)	O3—C13—C14—F11	4.6 (5)
C22—C23—C24—F20	178.7 (3)	O3—C13—C18—C17	175.4 (3)
C24—C19—C20—C21	−1.2 (5)	O3—C13—C18—F15	−3.7 (5)
C24—C19—C20—F16	177.1 (3)	O4—C19—C20—C21	−175.2 (3)
C24—C19—O4—P2	91.9 (3)	O4—C19—C20—F16	3.2 (5)
C25—C26—C27—C28	−0.4 (5)	O4—C19—C24—C23	175.5 (3)
C25—C26—C27—F22	178.4 (3)	O4—C19—C24—F20	−4.4 (5)
C25—O5—P3—N2	−131.9 (3)	O5—C25—C26—C27	−172.0 (3)
C25—O5—P3—N3	100.6 (3)	O5—C25—C26—F21	8.0 (5)
C25—O5—P3—O6	−15.7 (3)	O5—C25—C30—C29	173.3 (3)
C26—C25—C30—C29	−0.1 (5)	O5—C25—C30—F25	−5.8 (5)
C26—C25—C30—F25	−179.2 (3)	O6—C31—C32—C33	173.4 (3)
C26—C25—O5—P3	−63.1 (4)	O6—C31—C32—F26	−5.0 (5)
C26—C27—C28—C29	−1.3 (6)	O6—C31—C36—C35	−173.7 (4)
C26—C27—C28—F23	179.4 (3)	O6—C31—C36—F30	4.7 (5)
C27—C28—C29—C30	2.2 (6)	P1—N1—P2—N2	−7.2 (3)
C27—C28—C29—F24	179.9 (4)	P1—N1—P2—O3	−134.30 (18)
C28—C29—C30—C25	−1.5 (6)	P1—N1—P2—O4	119.40 (19)
C28—C29—C30—F25	177.6 (3)	P1—N3—P3—N2	−25.8 (2)
C30—C25—C26—C27	1.0 (5)	P1—N3—P3—O5	96.99 (19)
C30—C25—C26—F21	−179.0 (3)	P1—N3—P3—O6	−154.95 (16)
C30—C25—O5—P3	123.8 (3)	P2—N1—P1—N3	2.5 (3)
C31—C32—C33—C34	0.8 (6)	P2—N1—P1—O1	129.44 (18)
C31—C32—C33—F27	−179.2 (3)	P2—N1—P1—O2	−124.38 (18)
C31—O6—P3—N2	−57.9 (3)	P2—N2—P3—N3	21.2 (2)
C31—O6—P3—N3	73.2 (3)	P2—N2—P3—O5	−103.39 (19)
C31—O6—P3—O5	−170.6 (2)	P2—N2—P3—O6	149.87 (17)
C32—C31—C36—C35	−0.8 (6)	P3—N2—P2—N1	−5.2 (3)
C32—C31—C36—F30	177.6 (3)	P3—N2—P2—O3	123.00 (18)
C32—C31—O6—P3	94.2 (3)	P3—N2—P2—O4	−127.94 (17)
C32—C33—C34—C35	−1.8 (7)	P3—N3—P1—N1	14.5 (3)
C32—C33—C34—F28	−180.0 (4)	P3—N3—P1—O1	−108.68 (18)
C33—C34—C35—C36	1.6 (8)	P3—N3—P1—O2	142.74 (17)

### Hexakis[4-(trifluoromethyl)phenoxy]cyclotriphosphazene (2) Crystal data

**Table:**

C_42_H_24_F_18_N_3_O_6_P_3_	*Z* = 2
*M**_r_* = 1101.55	*F*(000) = 1104
Triclinic, *P*1	*D*_x_ = 1.599 Mg m^−^^3^
*a* = 13.4906 (11) Å	Mo *K*α radiation, λ = 0.71073 Å
*b* = 13.6573 (11) Å	Cell parameters from 4888 reflections
*c* = 14.9377 (13) Å	θ = 2.6–21.8°
α = 65.253 (2)°	µ = 0.25 mm^−^^1^
β = 68.874 (2)°	*T* = 298 K
γ = 71.715 (2)°	Cubical, colorless
*V* = 2288.0 (3) Å^3^	0.28 × 0.23 × 0.22 mm

### Hexakis[4-(trifluoromethyl)phenoxy]cyclotriphosphazene (2) Data collection

**Table:**

Bruker SMART CCD area detector diffractometer	11015 independent reflections
Radiation source: fine-focus sealed tube	6744 reflections with *I* > 2σ(*I*)
Graphite monochromator	*R*_int_ = 0.019
phi and ω scans	θ_max_ = 28.3°, θ_min_ = 1.7°
Absorption correction: multi-scan (SADABS; Bruker, 2001)	*h* = −17→16
*T*_min_ = 0.727, *T*_max_ = 0.9	*k* = −17→18
21657 measured reflections	*l* = −19→19

### Hexakis[4-(trifluoromethyl)phenoxy]cyclotriphosphazene (2) Refinement

**Table:**

Refinement on *F*^2^	Primary atom site location: structure-invariant direct methods
Least-squares matrix: full	Secondary atom site location: difference Fourier map
*R*[*F*^2^ > 2σ(*F*^2^)] = 0.062	Hydrogen site location: inferred from neighbouring sites
*wR*(*F*^2^) = 0.195	H-atom parameters constrained
*S* = 0.98	*w* = 1/[σ^2^(*F*_o_^2^) + (0.1123*P*)^2^ + 0.1708*P*] where *P* = (*F*_o_^2^ + 2*F*_c_^2^)/3
11015 reflections	(Δ/σ)_max_ < 0.001
817 parameters	Δρ_max_ = 0.32 e Å^−^^3^
462 restraints	Δρ_min_ = −0.27 e Å^−^^3^

### Hexakis[4-(trifluoromethyl)phenoxy]cyclotriphosphazene (2) Special details

**Table:**

Experimental. The data collection nominally covered a full sphere of reciprocal space by a combination of 4 sets of ω scans each set at different φ and/or 2θ angles and each scan (10 s exposure) covering -0.300° degrees in ω. The crystal to detector distance was 5.82 cm.
Geometry. All esds (except the esd in the dihedral angle between two l.s. planes) are estimated using the full covariance matrix. The cell esds are taken into account individually in the estimation of esds in distances, angles and torsion angles; correlations between esds in cell parameters are only used when they are defined by crystal symmetry. An approximate (isotropic) treatment of cell esds is used for estimating esds involving l.s. planes.
Refinement. Refinement of F^2^ against ALL reflections. The weighted R-factor wR and goodness of fit S are based on F^2^, conventional R-factors R are based on F, with F set to zero for negative F^2^. The threshold expression of F^2^ > 2sigma(F^2^) is used only for calculating R-factors(gt) etc. and is not relevant to the choice of reflections for refinement. R-factors based on F^2^ are statistically about twice as large as those based on F, and R- factors based on ALL data will be even larger.

### Hexakis[4-(trifluoromethyl)phenoxy]cyclotriphosphazene (2) Fractional atomic coordinates and isotropic or equivalent isotropic displacement parameters (Å^2^)

**Table:**

	*x*	*y*	*z*	*U*_iso_*/*U*_eq_	Occ. (<1)
P1	0.68414 (6)	0.84053 (6)	0.44920 (5)	0.0585 (2)	
P2	0.85174 (6)	0.67556 (6)	0.50300 (5)	0.0552 (2)	
P3	0.87918 (7)	0.88917 (7)	0.42221 (6)	0.0707 (3)	
F1A	0.4362 (12)	0.6424 (16)	0.1915 (7)	0.160 (5)	0.60 (2)
F2A	0.3227 (6)	0.6559 (12)	0.3199 (8)	0.145 (4)	0.60 (2)
F3A	0.4489 (17)	0.5186 (7)	0.3291 (19)	0.227 (8)	0.60 (2)
F4A	0.394 (3)	0.7186 (11)	0.9862 (16)	0.172 (7)	0.50 (4)
F5A	0.2833 (10)	0.866 (2)	0.9482 (15)	0.197 (7)	0.50 (4)
F6A	0.4337 (19)	0.864 (2)	0.9632 (17)	0.183 (8)	0.50 (4)
F7A	0.763 (2)	0.2483 (8)	0.3807 (10)	0.210 (9)	0.559 (18)
F8A	0.6714 (9)	0.3907 (12)	0.3012 (15)	0.193 (6)	0.559 (18)
F9A	0.8252 (12)	0.3395 (14)	0.2322 (9)	0.188 (6)	0.559 (18)
F10A	0.6296 (14)	0.6628 (14)	1.0167 (15)	0.220 (6)	0.58 (3)
F11A	0.6584 (13)	0.4891 (11)	1.0681 (7)	0.197 (6)	0.58 (3)
F12A	0.7777 (10)	0.5711 (19)	1.0454 (10)	0.225 (6)	0.58 (3)
F13A	1.000 (2)	0.6604 (14)	0.0487 (11)	0.153 (7)	0.41 (2)
F14A	1.1343 (9)	0.731 (3)	−0.0363 (17)	0.187 (10)	0.41 (2)
F15A	0.983 (2)	0.8130 (18)	−0.0613 (16)	0.187 (10)	0.41 (2)
F16A	0.7615 (13)	0.9015 (16)	0.9322 (8)	0.228 (9)	0.521 (18)
F17A	0.6226 (7)	0.9899 (11)	0.8914 (9)	0.144 (4)	0.521 (18)
F18A	0.7373 (15)	1.0756 (15)	0.8606 (11)	0.232 (11)	0.521 (18)
O1	0.65907 (17)	0.85648 (17)	0.34741 (14)	0.0702 (5)	
O2	0.56558 (16)	0.87706 (17)	0.51083 (15)	0.0704 (5)	
O3	0.91515 (14)	0.61655 (16)	0.42139 (14)	0.0636 (5)	
O4	0.86635 (16)	0.57561 (16)	0.60333 (14)	0.0662 (5)	
O5	0.9588 (2)	0.9303 (2)	0.31223 (17)	0.0940 (7)	
O6	0.9062 (2)	0.9631 (2)	0.46466 (17)	0.0881 (7)	
N1	0.72831 (17)	0.71653 (18)	0.50653 (16)	0.0560 (5)	
N2	0.91743 (19)	0.7639 (2)	0.48024 (19)	0.0666 (6)	
N3	0.7578 (2)	0.9251 (2)	0.41827 (19)	0.0720 (7)	
C1	0.6004 (2)	0.7960 (3)	0.3373 (2)	0.0631 (7)	
C2	0.6003 (3)	0.8158 (3)	0.2394 (2)	0.0760 (8)	
H2	0.6376	0.8676	0.1846	0.091*	
C3	0.5447 (3)	0.7587 (3)	0.2226 (2)	0.0848 (10)	
H3	0.5452	0.7717	0.1562	0.102*	
C4	0.4886 (3)	0.6825 (3)	0.3033 (3)	0.0785 (9)	
C5	0.4888 (3)	0.6644 (3)	0.4011 (2)	0.0811 (9)	
H5	0.4503	0.6137	0.4560	0.097*	
C6	0.5446 (3)	0.7195 (3)	0.4194 (2)	0.0769 (9)	
H6	0.5449	0.7058	0.4857	0.092*	
C7	0.4253 (4)	0.6231 (5)	0.2866 (3)	0.1041 (12)	
C8	0.5285 (2)	0.8581 (2)	0.6156 (2)	0.0616 (7)	
C9	0.5845 (3)	0.8670 (5)	0.6688 (3)	0.1203 (17)	
H9	0.6539	0.8823	0.6371	0.144*	
C10	0.5384 (4)	0.8532 (5)	0.7715 (3)	0.1295 (18)	
H10	0.5779	0.8590	0.8082	0.155*	
C11	0.4390 (3)	0.8320 (3)	0.8189 (3)	0.0821 (9)	
C12	0.3834 (4)	0.8241 (4)	0.7636 (3)	0.1165 (15)	
H12	0.3140	0.8088	0.7953	0.140*	
C13	0.4271 (3)	0.8381 (4)	0.6617 (3)	0.1033 (13)	
H13	0.3871	0.8339	0.6247	0.124*	
C14	0.3876 (4)	0.8233 (5)	0.9270 (3)	0.1174 (14)	
C15	0.8713 (2)	0.5499 (2)	0.40165 (19)	0.0560 (6)	
C16	0.8619 (2)	0.4457 (2)	0.4678 (2)	0.0640 (7)	
H16	0.8793	0.4197	0.5296	0.077*	
C17	0.8263 (2)	0.3799 (3)	0.4419 (2)	0.0688 (7)	
H17	0.8195	0.3088	0.4865	0.083*	
C18	0.8004 (3)	0.4186 (3)	0.3498 (2)	0.0697 (7)	
C19	0.8091 (3)	0.5248 (3)	0.2851 (2)	0.0770 (9)	
H19	0.7911	0.5518	0.2234	0.092*	
C20	0.8438 (3)	0.5907 (3)	0.3109 (2)	0.0716 (8)	
H20	0.8488	0.6625	0.2675	0.086*	
C21	0.7667 (4)	0.3459 (4)	0.3208 (3)	0.1023 (12)	
C22	0.8220 (2)	0.5822 (2)	0.7016 (2)	0.0615 (7)	
C23	0.8860 (3)	0.5992 (3)	0.7438 (3)	0.0923 (11)	
H23	0.9556	0.6118	0.7064	0.111*	
C24	0.8460 (4)	0.5973 (4)	0.8433 (3)	0.1145 (14)	
H24	0.8890	0.6081	0.8737	0.137*	
C25	0.7440 (5)	0.5799 (4)	0.8972 (3)	0.1115 (14)	
C26	0.6821 (4)	0.5634 (4)	0.8531 (3)	0.1285 (17)	
H26	0.6123	0.5513	0.8901	0.154*	
C27	0.7209 (3)	0.5642 (4)	0.7538 (3)	0.0996 (12)	
H27	0.6782	0.5526	0.7236	0.119*	
C28	0.7003 (7)	0.5779 (6)	1.0037 (4)	0.177 (3)	
C29	0.9745 (3)	0.8835 (3)	0.2388 (2)	0.0810 (9)	
C30	1.0614 (3)	0.8001 (4)	0.2282 (3)	0.0992 (12)	
H30	1.1073	0.7725	0.2706	0.119*	
C31	1.0793 (3)	0.7581 (4)	0.1533 (3)	0.1006 (12)	
H31	1.1376	0.7011	0.1454	0.121*	
C32	1.0123 (3)	0.7991 (3)	0.0902 (3)	0.0864 (10)	
C33	0.9258 (3)	0.8820 (3)	0.1029 (3)	0.0924 (10)	
H33	0.8796	0.9099	0.0607	0.111*	
C34	0.9067 (3)	0.9247 (3)	0.1780 (3)	0.0898 (10)	
H34	0.8480	0.9811	0.1866	0.108*	
C35	1.0318 (4)	0.7530 (4)	0.0104 (3)	0.1108 (13)	
C36	0.8587 (3)	0.9643 (3)	0.5638 (2)	0.0735 (8)	
C37	0.7719 (4)	1.0433 (3)	0.5824 (3)	0.1078 (13)	
H37	0.7421	1.0944	0.5294	0.129*	
C38	0.7283 (4)	1.0481 (4)	0.6782 (4)	0.1182 (15)	
H38	0.6672	1.1010	0.6908	0.142*	
C39	0.7728 (3)	0.9766 (3)	0.7557 (3)	0.0897 (10)	
C40	0.8609 (4)	0.8990 (4)	0.7358 (3)	0.1053 (12)	
H40	0.8920	0.8490	0.7882	0.126*	
C41	0.9049 (3)	0.8930 (4)	0.6389 (3)	0.0967 (11)	
H41	0.9659	0.8403	0.6257	0.116*	
C42	0.7262 (5)	0.9837 (5)	0.8593 (4)	0.1269 (16)	
F1B	0.3334 (15)	0.6780 (15)	0.268 (2)	0.214 (10)	0.40 (2)
F3B	0.4773 (17)	0.580 (2)	0.2181 (17)	0.163 (9)	0.40 (2)
F2B	0.3929 (15)	0.5385 (14)	0.3684 (6)	0.137 (5)	0.40 (2)
F8B	0.730 (2)	0.3875 (10)	0.2449 (11)	0.182 (9)	0.441 (18)
F7B	0.6902 (10)	0.2962 (12)	0.3998 (9)	0.134 (5)	0.441 (18)
F9B	0.8426 (8)	0.2602 (12)	0.3094 (17)	0.159 (7)	0.441 (18)
F14B	1.1116 (15)	0.6706 (11)	0.0104 (12)	0.194 (6)	0.59 (2)
F15B	1.0467 (16)	0.8241 (9)	−0.0802 (6)	0.166 (6)	0.59 (2)
F13B	0.9472 (11)	0.7161 (17)	0.0203 (13)	0.179 (6)	0.59 (2)
F18B	0.6521 (17)	1.0665 (18)	0.8675 (12)	0.266 (13)	0.479 (18)
F16B	0.7994 (9)	0.9847 (11)	0.8964 (8)	0.142 (4)	0.479 (18)
F17B	0.688 (2)	0.8961 (17)	0.9256 (10)	0.250 (14)	0.479 (18)
F10B	0.685 (3)	0.6819 (12)	1.0010 (18)	0.264 (13)	0.42 (3)
F12B	0.773 (2)	0.523 (3)	1.055 (2)	0.311 (15)	0.42 (3)
F11B	0.605 (2)	0.542 (3)	1.0475 (17)	0.282 (11)	0.42 (3)
F5B	0.3040 (18)	0.9045 (19)	0.9332 (13)	0.182 (7)	0.50 (4)
F4B	0.351 (3)	0.7310 (15)	0.9857 (18)	0.174 (7)	0.50 (4)
F6B	0.4543 (15)	0.825 (3)	0.9713 (13)	0.170 (7)	0.50 (4)

### Hexakis[4-(trifluoromethyl)phenoxy]cyclotriphosphazene (2) Atomic displacement parameters (Å^2^)

**Table:**

	*U*^11^	*U*^22^	*U*^33^	*U*^12^	*U*^13^	*U*^23^
P1	0.0639 (4)	0.0609 (4)	0.0499 (4)	−0.0100 (3)	−0.0165 (3)	−0.0186 (3)
P2	0.0554 (4)	0.0635 (4)	0.0488 (4)	−0.0100 (3)	−0.0114 (3)	−0.0246 (3)
P3	0.0831 (6)	0.0789 (6)	0.0596 (4)	−0.0359 (4)	−0.0091 (4)	−0.0265 (4)
F1A	0.160 (8)	0.289 (15)	0.108 (4)	−0.113 (9)	−0.007 (4)	−0.113 (6)
F2A	0.085 (4)	0.265 (10)	0.136 (6)	−0.079 (5)	0.007 (4)	−0.114 (6)
F3A	0.282 (17)	0.135 (5)	0.348 (19)	−0.057 (7)	−0.192 (16)	−0.060 (9)
F4A	0.235 (19)	0.197 (7)	0.067 (6)	−0.072 (8)	−0.028 (10)	−0.017 (5)
F5A	0.106 (6)	0.326 (19)	0.093 (6)	−0.021 (7)	0.023 (4)	−0.069 (10)
F6A	0.184 (12)	0.292 (17)	0.140 (11)	−0.100 (12)	0.029 (8)	−0.159 (13)
F7A	0.44 (3)	0.103 (7)	0.163 (10)	−0.123 (12)	−0.179 (13)	0.015 (7)
F8A	0.191 (7)	0.216 (10)	0.268 (16)	−0.050 (6)	−0.137 (9)	−0.102 (11)
F9A	0.304 (12)	0.172 (12)	0.142 (7)	−0.084 (10)	−0.030 (8)	−0.101 (8)
F10A	0.260 (11)	0.235 (9)	0.117 (8)	−0.023 (8)	0.041 (6)	−0.111 (8)
F11A	0.251 (12)	0.247 (9)	0.047 (3)	−0.070 (8)	−0.019 (5)	−0.009 (5)
F12A	0.318 (10)	0.315 (19)	0.070 (5)	−0.098 (9)	−0.029 (7)	−0.084 (8)
F13A	0.234 (18)	0.148 (10)	0.112 (7)	−0.082 (12)	−0.029 (9)	−0.059 (6)
F14A	0.125 (7)	0.33 (3)	0.152 (14)	−0.059 (10)	0.029 (7)	−0.173 (17)
F15A	0.28 (2)	0.190 (14)	0.132 (14)	0.022 (13)	−0.120 (16)	−0.083 (9)
F16A	0.236 (15)	0.33 (2)	0.087 (6)	−0.027 (13)	−0.049 (8)	−0.061 (10)
F17A	0.136 (5)	0.196 (10)	0.123 (6)	−0.061 (6)	0.017 (4)	−0.098 (7)
F18A	0.29 (2)	0.318 (18)	0.198 (12)	−0.195 (17)	0.067 (11)	−0.201 (13)
O1	0.0840 (13)	0.0768 (13)	0.0509 (10)	−0.0240 (11)	−0.0214 (9)	−0.0138 (9)
O2	0.0665 (12)	0.0786 (13)	0.0612 (11)	0.0021 (10)	−0.0210 (9)	−0.0279 (10)
O3	0.0581 (11)	0.0783 (13)	0.0607 (11)	−0.0154 (9)	−0.0065 (8)	−0.0361 (10)
O4	0.0727 (12)	0.0677 (12)	0.0575 (11)	−0.0011 (9)	−0.0217 (9)	−0.0262 (9)
O5	0.1126 (18)	0.1132 (18)	0.0669 (13)	−0.0661 (15)	0.0019 (12)	−0.0309 (13)
O6	0.1099 (17)	0.0967 (16)	0.0781 (14)	−0.0568 (14)	−0.0081 (12)	−0.0366 (13)
N1	0.0575 (12)	0.0606 (13)	0.0489 (11)	−0.0162 (10)	−0.0093 (9)	−0.0185 (10)
N2	0.0619 (14)	0.0812 (16)	0.0677 (14)	−0.0205 (12)	−0.0142 (11)	−0.0335 (12)
N3	0.0925 (18)	0.0583 (14)	0.0669 (15)	−0.0211 (13)	−0.0246 (13)	−0.0157 (12)
C1	0.0589 (16)	0.0769 (19)	0.0531 (14)	−0.0079 (14)	−0.0158 (12)	−0.0249 (14)
C2	0.081 (2)	0.096 (2)	0.0493 (15)	−0.0209 (17)	−0.0143 (14)	−0.0228 (16)
C3	0.079 (2)	0.124 (3)	0.0612 (18)	−0.019 (2)	−0.0161 (16)	−0.0440 (19)
C4	0.0644 (18)	0.109 (3)	0.0738 (19)	−0.0169 (17)	−0.0128 (15)	−0.0473 (19)
C5	0.078 (2)	0.106 (3)	0.0636 (18)	−0.0318 (19)	−0.0124 (15)	−0.0278 (18)
C6	0.081 (2)	0.103 (2)	0.0524 (15)	−0.0294 (18)	−0.0194 (14)	−0.0226 (16)
C7	0.095 (3)	0.153 (4)	0.091 (3)	−0.043 (3)	−0.017 (2)	−0.061 (3)
C8	0.0598 (16)	0.0628 (16)	0.0622 (15)	−0.0025 (13)	−0.0154 (12)	−0.0288 (14)
C9	0.085 (2)	0.225 (5)	0.085 (2)	−0.069 (3)	0.0042 (19)	−0.079 (3)
C10	0.101 (3)	0.235 (6)	0.092 (3)	−0.066 (3)	−0.003 (2)	−0.087 (3)
C11	0.082 (2)	0.094 (2)	0.0700 (19)	−0.0173 (18)	−0.0098 (16)	−0.0356 (18)
C12	0.098 (3)	0.173 (4)	0.095 (3)	−0.073 (3)	0.012 (2)	−0.060 (3)
C13	0.096 (3)	0.151 (4)	0.088 (2)	−0.055 (3)	−0.008 (2)	−0.055 (3)
C14	0.110 (3)	0.157 (4)	0.080 (3)	−0.029 (3)	−0.005 (2)	−0.050 (3)
C15	0.0507 (14)	0.0674 (17)	0.0497 (13)	−0.0070 (12)	−0.0079 (11)	−0.0277 (13)
C16	0.0671 (17)	0.0760 (19)	0.0465 (14)	−0.0122 (14)	−0.0157 (12)	−0.0195 (13)
C17	0.081 (2)	0.0674 (18)	0.0551 (15)	−0.0225 (15)	−0.0184 (14)	−0.0124 (14)
C18	0.0765 (19)	0.078 (2)	0.0614 (16)	−0.0232 (15)	−0.0194 (14)	−0.0234 (15)
C19	0.102 (2)	0.081 (2)	0.0543 (16)	−0.0210 (18)	−0.0329 (16)	−0.0164 (15)
C20	0.094 (2)	0.0670 (18)	0.0544 (16)	−0.0190 (16)	−0.0221 (15)	−0.0163 (14)
C21	0.140 (4)	0.100 (3)	0.088 (3)	−0.046 (3)	−0.043 (3)	−0.026 (2)
C22	0.0718 (18)	0.0610 (16)	0.0487 (14)	−0.0078 (13)	−0.0186 (12)	−0.0173 (12)
C23	0.085 (2)	0.130 (3)	0.076 (2)	−0.023 (2)	−0.0173 (17)	−0.051 (2)
C24	0.144 (4)	0.140 (4)	0.077 (2)	−0.016 (3)	−0.039 (2)	−0.053 (3)
C25	0.147 (4)	0.103 (3)	0.0523 (19)	0.003 (3)	−0.016 (2)	−0.0240 (19)
C26	0.109 (3)	0.160 (5)	0.073 (3)	−0.046 (3)	0.016 (2)	−0.019 (3)
C27	0.093 (3)	0.135 (3)	0.070 (2)	−0.052 (2)	−0.0109 (18)	−0.022 (2)
C28	0.246 (7)	0.166 (5)	0.063 (3)	0.008 (5)	−0.018 (4)	−0.040 (4)
C29	0.088 (2)	0.099 (2)	0.0555 (17)	−0.047 (2)	0.0005 (15)	−0.0222 (17)
C30	0.078 (2)	0.142 (4)	0.070 (2)	−0.027 (2)	−0.0080 (17)	−0.034 (2)
C31	0.086 (2)	0.120 (3)	0.074 (2)	−0.012 (2)	−0.0026 (18)	−0.035 (2)
C32	0.093 (2)	0.097 (3)	0.0602 (18)	−0.029 (2)	−0.0028 (17)	−0.0261 (18)
C33	0.107 (3)	0.100 (3)	0.067 (2)	−0.019 (2)	−0.0254 (19)	−0.0249 (19)
C34	0.101 (3)	0.088 (2)	0.072 (2)	−0.016 (2)	−0.0161 (19)	−0.0269 (18)
C35	0.137 (4)	0.112 (3)	0.080 (3)	−0.031 (3)	−0.012 (3)	−0.039 (2)
C36	0.089 (2)	0.072 (2)	0.0759 (19)	−0.0342 (17)	−0.0187 (17)	−0.0303 (16)
C37	0.146 (4)	0.082 (2)	0.095 (3)	0.005 (2)	−0.049 (3)	−0.036 (2)
C38	0.142 (4)	0.106 (3)	0.111 (3)	0.018 (3)	−0.047 (3)	−0.060 (3)
C39	0.099 (3)	0.108 (3)	0.088 (2)	−0.025 (2)	−0.0249 (19)	−0.054 (2)
C40	0.120 (3)	0.119 (3)	0.096 (3)	−0.008 (3)	−0.054 (2)	−0.044 (2)
C41	0.094 (3)	0.114 (3)	0.104 (3)	−0.002 (2)	−0.042 (2)	−0.058 (2)
C42	0.141 (4)	0.161 (5)	0.106 (3)	−0.038 (4)	−0.024 (3)	−0.072 (3)
F1B	0.167 (12)	0.232 (12)	0.33 (3)	0.020 (11)	−0.176 (16)	−0.135 (15)
F3B	0.156 (12)	0.238 (19)	0.158 (12)	−0.112 (12)	0.047 (10)	−0.148 (13)
F2B	0.168 (11)	0.200 (11)	0.090 (6)	−0.117 (9)	−0.015 (5)	−0.049 (5)
F8B	0.37 (3)	0.116 (9)	0.146 (10)	−0.088 (12)	−0.183 (15)	0.000 (7)
F7B	0.155 (8)	0.147 (10)	0.138 (7)	−0.097 (7)	−0.006 (5)	−0.064 (7)
F9B	0.174 (8)	0.137 (11)	0.224 (18)	−0.012 (7)	−0.062 (9)	−0.122 (13)
F14B	0.259 (12)	0.146 (9)	0.168 (11)	0.043 (8)	−0.063 (9)	−0.099 (8)
F15B	0.294 (15)	0.135 (6)	0.060 (3)	−0.081 (8)	−0.006 (6)	−0.032 (3)
F13B	0.226 (10)	0.218 (14)	0.144 (10)	−0.109 (10)	−0.013 (7)	−0.094 (10)
F18B	0.282 (19)	0.34 (2)	0.183 (15)	0.165 (17)	−0.127 (15)	−0.200 (17)
F16B	0.185 (8)	0.184 (10)	0.105 (6)	−0.033 (7)	−0.067 (6)	−0.072 (7)
F17B	0.39 (3)	0.30 (2)	0.102 (8)	−0.23 (2)	0.049 (14)	−0.094 (11)
F10B	0.42 (3)	0.224 (10)	0.096 (9)	−0.018 (13)	0.003 (17)	−0.101 (9)
F12B	0.50 (2)	0.31 (3)	0.124 (12)	0.02 (2)	−0.163 (17)	−0.077 (14)
F11B	0.356 (18)	0.27 (3)	0.084 (11)	−0.09 (2)	0.098 (11)	−0.052 (13)
F5B	0.174 (11)	0.214 (11)	0.104 (8)	0.054 (9)	−0.005 (7)	−0.098 (8)
F4B	0.191 (15)	0.198 (9)	0.090 (8)	−0.080 (9)	0.024 (8)	−0.031 (6)
F6B	0.152 (8)	0.299 (18)	0.079 (5)	−0.060 (9)	−0.011 (5)	−0.089 (9)

### Hexakis[4-(trifluoromethyl)phenoxy]cyclotriphosphazene (2) Geometric parameters (Å, º)

**Table:**

P1—O1	1.588 (2)	C13—H13	0.9300
P1—O2	1.574 (2)	C14—F5B	1.317 (10)
P1—N1	1.574 (2)	C14—F4B	1.325 (12)
P1—N3	1.574 (3)	C14—F6B	1.302 (11)
P2—O3	1.5829 (18)	C15—C16	1.364 (4)
P2—O4	1.575 (2)	C15—C20	1.373 (4)
P2—N1	1.570 (2)	C16—H16	0.9300
P2—N2	1.570 (2)	C16—C17	1.373 (4)
P3—O5	1.582 (2)	C17—H17	0.9300
P3—O6	1.577 (2)	C17—C18	1.383 (4)
P3—N2	1.573 (3)	C18—C19	1.380 (4)
P3—N3	1.571 (3)	C18—C21	1.471 (5)
F1A—C7	1.291 (8)	C19—H19	0.9300
F2A—C7	1.290 (8)	C19—C20	1.365 (4)
F3A—C7	1.281 (9)	C20—H20	0.9300
F4A—C14	1.324 (12)	C21—F8B	1.244 (9)
F5A—C14	1.319 (12)	C21—F7B	1.346 (9)
F6A—C14	1.304 (12)	C21—F9B	1.318 (9)
F7A—C21	1.261 (7)	C22—C23	1.356 (4)
F8A—C21	1.320 (9)	C22—C27	1.345 (5)
F9A—C21	1.297 (8)	C23—H23	0.9300
F10A—C28	1.285 (12)	C23—C24	1.378 (5)
F11A—C28	1.333 (10)	C24—H24	0.9300
F12A—C28	1.358 (12)	C24—C25	1.358 (7)
F13A—C35	1.290 (11)	C25—C26	1.350 (7)
F14A—C35	1.307 (10)	C25—C28	1.477 (6)
F15A—C35	1.297 (11)	C26—H26	0.9300
F16A—C42	1.300 (11)	C26—C27	1.380 (6)
F17A—C42	1.291 (9)	C27—H27	0.9300
F18A—C42	1.317 (10)	C28—F10B	1.355 (13)
O1—C1	1.391 (3)	C28—F12B	1.307 (14)
O2—C8	1.394 (3)	C28—F11B	1.351 (14)
O3—C15	1.398 (3)	C29—C30	1.369 (5)
O4—C22	1.401 (3)	C29—C34	1.350 (5)
O5—C29	1.412 (4)	C30—H30	0.9300
O6—C36	1.391 (4)	C30—C31	1.377 (6)
C1—C2	1.371 (4)	C31—H31	0.9300
C1—C6	1.384 (4)	C31—C32	1.368 (5)
C2—H2	0.9300	C32—C33	1.368 (5)
C2—C3	1.379 (5)	C32—C35	1.478 (5)
C3—H3	0.9300	C33—H33	0.9300
C3—C4	1.376 (5)	C33—C34	1.382 (5)
C4—C5	1.376 (4)	C34—H34	0.9300
C4—C7	1.487 (5)	C35—F14B	1.291 (10)
C5—H5	0.9300	C35—F15B	1.282 (8)
C5—C6	1.372 (4)	C35—F13B	1.325 (9)
C6—H6	0.9300	C36—C37	1.356 (5)
C7—F1B	1.289 (11)	C36—C41	1.342 (5)
C7—F3B	1.272 (10)	C37—H37	0.9300
C7—F2B	1.330 (11)	C37—C38	1.359 (6)
C8—C9	1.337 (5)	C38—H38	0.9300
C8—C13	1.348 (4)	C38—C39	1.357 (6)
C9—H9	0.9300	C39—C40	1.358 (6)
C9—C10	1.385 (5)	C39—C42	1.478 (5)
C10—H10	0.9300	C40—H40	0.9300
C10—C11	1.331 (5)	C40—C41	1.379 (5)
C11—C12	1.354 (5)	C41—H41	0.9300
C11—C14	1.478 (5)	C42—F18B	1.269 (10)
C12—H12	0.9300	C42—F16B	1.301 (8)
C12—C13	1.371 (5)	C42—F17B	1.304 (11)
			
O2—P1—O1	98.79 (11)	F9A—C21—C18	112.4 (6)
O2—P1—N1	109.73 (12)	F9A—C21—F7B	137.9 (6)
O2—P1—N3	111.74 (13)	F9A—C21—F9B	56.9 (7)
N1—P1—O1	111.78 (12)	F8B—C21—F7A	122.5 (7)
N1—P1—N3	116.95 (13)	F8B—C21—F8A	42.3 (9)
N3—P1—O1	106.31 (12)	F8B—C21—F9A	58.0 (8)
O4—P2—O3	99.42 (11)	F8B—C21—C18	118.0 (6)
N1—P2—O3	111.82 (11)	F8B—C21—F7B	106.4 (10)
N1—P2—O4	109.86 (11)	F8B—C21—F9B	107.4 (9)
N2—P2—O3	106.23 (12)	F7B—C21—C18	109.2 (6)
N2—P2—O4	110.87 (12)	F9B—C21—F8A	135.6 (7)
N2—P2—N1	117.16 (13)	F9B—C21—C18	113.4 (6)
O6—P3—O5	94.47 (12)	F9B—C21—F7B	100.8 (8)
N2—P3—O5	111.37 (15)	C23—C22—O4	118.2 (3)
N2—P3—O6	110.74 (13)	C27—C22—O4	119.7 (3)
N3—P3—O5	111.60 (15)	C27—C22—C23	121.9 (3)
N3—P3—O6	110.74 (14)	C22—C23—H23	120.7
N3—P3—N2	115.92 (13)	C22—C23—C24	118.7 (4)
C1—O1—P1	126.59 (18)	C24—C23—H23	120.7
C8—O2—P1	127.04 (18)	C23—C24—H24	119.8
C15—O3—P2	123.62 (15)	C25—C24—C23	120.4 (4)
C22—O4—P2	123.54 (17)	C25—C24—H24	119.8
C29—O5—P3	120.19 (19)	C24—C25—C28	120.8 (6)
C36—O6—P3	123.21 (19)	C26—C25—C24	119.6 (4)
P2—N1—P1	120.25 (14)	C26—C25—C28	119.6 (6)
P2—N2—P3	121.42 (15)	C25—C26—H26	119.6
P3—N3—P1	122.96 (16)	C25—C26—C27	120.9 (4)
C2—C1—O1	116.0 (3)	C27—C26—H26	119.6
C2—C1—C6	120.5 (3)	C22—C27—C26	118.5 (4)
C6—C1—O1	123.4 (2)	C22—C27—H27	120.7
C1—C2—H2	120.1	C26—C27—H27	120.7
C1—C2—C3	119.7 (3)	F10A—C28—F11A	107.9 (10)
C3—C2—H2	120.1	F10A—C28—F12A	104.0 (12)
C2—C3—H3	119.7	F10A—C28—C25	115.5 (10)
C4—C3—C2	120.5 (3)	F10A—C28—F10B	35.0 (17)
C4—C3—H3	119.7	F10A—C28—F12B	125 (2)
C3—C4—C5	118.9 (3)	F10A—C28—F11B	75.9 (12)
C3—C4—C7	121.1 (3)	F11A—C28—F12A	102.1 (10)
C5—C4—C7	120.0 (3)	F11A—C28—C25	113.2 (8)
C4—C5—H5	119.3	F11A—C28—F10B	135.7 (13)
C6—C5—C4	121.4 (3)	F11A—C28—F11B	38.3 (14)
C6—C5—H5	119.3	F12A—C28—C25	113.0 (8)
C1—C6—H6	120.6	F10B—C28—F12A	77.0 (19)
C5—C6—C1	118.8 (3)	F10B—C28—C25	107.3 (11)
C5—C6—H6	120.6	F12B—C28—F11A	78.3 (18)
F1A—C7—C4	114.1 (7)	F12B—C28—F12A	28 (2)
F1A—C7—F2B	129.2 (9)	F12B—C28—C25	110.9 (13)
F2A—C7—F1A	102.6 (8)	F12B—C28—F10B	103.6 (16)
F2A—C7—C4	110.8 (6)	F12B—C28—F11B	113.7 (15)
F2A—C7—F2B	75.3 (9)	F11B—C28—F12A	130.9 (15)
F3A—C7—F1A	106.9 (9)	F11B—C28—C25	110.6 (10)
F3A—C7—F2A	109.0 (10)	F11B—C28—F10B	110.4 (14)
F3A—C7—C4	112.8 (6)	C30—C29—O5	118.7 (3)
F3A—C7—F1B	125.6 (12)	C34—C29—O5	119.6 (4)
F3A—C7—F2B	36.6 (10)	C34—C29—C30	121.7 (4)
F1B—C7—F1A	73.2 (11)	C29—C30—H30	120.7
F1B—C7—F2A	30.5 (13)	C29—C30—C31	118.6 (4)
F1B—C7—C4	116.1 (9)	C31—C30—H30	120.7
F1B—C7—F2B	100.1 (11)	C30—C31—H31	119.6
F3B—C7—F1A	40.2 (11)	C32—C31—C30	120.8 (4)
F3B—C7—F2A	130.7 (11)	C32—C31—H31	119.6
F3B—C7—F3A	70.5 (10)	C31—C32—C35	120.4 (4)
F3B—C7—C4	114.3 (9)	C33—C32—C31	119.3 (4)
F3B—C7—F1B	107.9 (12)	C33—C32—C35	120.3 (4)
F3B—C7—F2B	103.1 (11)	C32—C33—H33	119.8
F2B—C7—C4	113.7 (6)	C32—C33—C34	120.5 (4)
C9—C8—O2	123.6 (3)	C34—C33—H33	119.8
C9—C8—C13	119.7 (3)	C29—C34—C33	119.2 (4)
C13—C8—O2	116.5 (3)	C29—C34—H34	120.4
C8—C9—H9	120.2	C33—C34—H34	120.4
C8—C9—C10	119.6 (4)	F13A—C35—F14A	104.9 (11)
C10—C9—H9	120.2	F13A—C35—F15A	104.7 (11)
C9—C10—H10	119.1	F13A—C35—C32	111.5 (8)
C11—C10—C9	121.7 (4)	F13A—C35—F14B	67.5 (9)
C11—C10—H10	119.1	F13A—C35—F13B	41.9 (8)
C10—C11—C12	117.9 (3)	F14A—C35—C32	113.0 (8)
C10—C11—C14	121.7 (4)	F14A—C35—F13B	131.9 (10)
C12—C11—C14	120.4 (4)	F15A—C35—F14A	105.7 (10)
C11—C12—H12	119.3	F15A—C35—C32	115.9 (10)
C11—C12—C13	121.4 (4)	F15A—C35—F13B	67.0 (10)
C13—C12—H12	119.3	F14B—C35—F14A	40.0 (9)
C8—C13—C12	119.7 (4)	F14B—C35—F15A	128.0 (11)
C8—C13—H13	120.1	F14B—C35—C32	114.3 (7)
C12—C13—H13	120.1	F14B—C35—F13B	105.0 (9)
F4A—C14—C11	109.8 (11)	F15B—C35—F13A	131.7 (9)
F4A—C14—F4B	24.1 (18)	F15B—C35—F14A	72.7 (11)
F5A—C14—F4A	104.6 (12)	F15B—C35—F15A	38.1 (11)
F5A—C14—C11	115.0 (9)	F15B—C35—C32	113.4 (7)
F5A—C14—F4B	81.8 (15)	F15B—C35—F14B	107.8 (8)
F6A—C14—F4A	104.4 (13)	F15B—C35—F13B	103.1 (9)
F6A—C14—F5A	107.2 (12)	F13B—C35—C32	112.3 (6)
F6A—C14—C11	114.7 (10)	C37—C36—O6	119.8 (3)
F6A—C14—F5B	88.1 (15)	C41—C36—O6	119.6 (3)
F6A—C14—F4B	119.4 (19)	C41—C36—C37	120.3 (3)
F5B—C14—F4A	127.7 (15)	C36—C37—H37	119.9
F5B—C14—F5A	26.0 (16)	C36—C37—C38	120.1 (4)
F5B—C14—C11	110.0 (8)	C38—C37—H37	119.9
F5B—C14—F4B	106.6 (11)	C37—C38—H38	119.6
F4B—C14—C11	114.1 (12)	C39—C38—C37	120.8 (4)
F6B—C14—F4A	84.9 (16)	C39—C38—H38	119.6
F6B—C14—F5A	123.9 (14)	C38—C39—C40	118.5 (4)
F6B—C14—F6A	22.5 (19)	C38—C39—C42	120.5 (4)
F6B—C14—C11	112.6 (9)	C40—C39—C42	121.0 (4)
F6B—C14—F5B	109.0 (11)	C39—C40—H40	119.5
F6B—C14—F4B	104.1 (13)	C39—C40—C41	121.0 (4)
C16—C15—O3	120.9 (2)	C41—C40—H40	119.5
C16—C15—C20	121.3 (3)	C36—C41—C40	119.3 (4)
C20—C15—O3	117.6 (3)	C36—C41—H41	120.4
C15—C16—H16	120.4	C40—C41—H41	120.4
C15—C16—C17	119.2 (3)	F16A—C42—F18A	108.9 (10)
C17—C16—H16	120.4	F16A—C42—C39	116.2 (8)
C16—C17—H17	119.8	F16A—C42—F16B	56.1 (8)
C16—C17—C18	120.4 (3)	F16A—C42—F17B	47.8 (9)
C18—C17—H17	119.8	F17A—C42—F16A	103.3 (9)
C17—C18—C21	120.0 (3)	F17A—C42—F18A	102.6 (8)
C19—C18—C17	119.2 (3)	F17A—C42—C39	113.3 (6)
C19—C18—C21	120.8 (3)	F17A—C42—F16B	134.5 (7)
C18—C19—H19	119.7	F17A—C42—F17B	61.3 (11)
C20—C19—C18	120.5 (3)	F18A—C42—C39	111.3 (7)
C20—C19—H19	119.7	F18B—C42—F16A	127.5 (11)
C15—C20—H20	120.3	F18B—C42—F17A	51.2 (12)
C19—C20—C15	119.4 (3)	F18B—C42—F18A	53.2 (9)
C19—C20—H20	120.3	F18B—C42—C39	116.1 (7)
F7A—C21—F8A	107.5 (9)	F18B—C42—F16B	105.9 (10)
F7A—C21—F9A	106.0 (8)	F18B—C42—F17B	107.5 (12)
F7A—C21—C18	118.9 (5)	F16B—C42—F18A	58.6 (8)
F7A—C21—F7B	45.6 (8)	F16B—C42—C39	112.1 (6)
F7A—C21—F9B	55.7 (7)	F16B—C42—F17B	102.4 (11)
F8A—C21—C18	110.4 (6)	F17B—C42—F18A	136.9 (9)
F8A—C21—F7B	70.6 (8)	F17B—C42—C39	111.7 (8)
F9A—C21—F8A	99.8 (8)		
			
P1—O1—C1—C2	−172.4 (2)	C10—C11—C14—F6B	−8.2 (17)
P1—O1—C1—C6	7.5 (4)	C11—C12—C13—C8	1.4 (8)
P1—O2—C8—C9	41.2 (5)	C12—C11—C14—F4A	82.5 (16)
P1—O2—C8—C13	−144.0 (3)	C12—C11—C14—F5A	−35.2 (17)
P2—O3—C15—C16	−75.8 (3)	C12—C11—C14—F6A	−160.3 (16)
P2—O3—C15—C20	107.8 (3)	C12—C11—C14—F5B	−62.9 (17)
P2—O4—C22—C23	−96.7 (3)	C12—C11—C14—F4B	56.9 (17)
P2—O4—C22—C27	87.9 (4)	C12—C11—C14—F6B	175.3 (16)
P3—O5—C29—C30	−95.9 (4)	C13—C8—C9—C10	1.4 (7)
P3—O5—C29—C34	86.9 (4)	C14—C11—C12—C13	176.2 (5)
P3—O6—C36—C37	−96.7 (4)	C15—C16—C17—C18	0.1 (4)
P3—O6—C36—C41	89.0 (4)	C16—C15—C20—C19	−1.7 (4)
O1—P1—O2—C8	164.1 (2)	C16—C17—C18—C19	−1.1 (5)
O1—P1—N1—P2	105.08 (16)	C16—C17—C18—C21	177.5 (3)
O1—P1—N3—P3	−113.22 (18)	C17—C18—C19—C20	0.7 (5)
O1—C1—C2—C3	179.5 (3)	C17—C18—C21—F7A	−0.7 (15)
O1—C1—C6—C5	179.8 (3)	C17—C18—C21—F8A	124.2 (10)
O2—P1—O1—C1	−67.0 (2)	C17—C18—C21—F9A	−125.3 (10)
O2—P1—N1—P2	−146.37 (14)	C17—C18—C21—F8B	170.1 (14)
O2—P1—N3—P3	140.03 (17)	C17—C18—C21—F7B	48.5 (9)
O2—C8—C9—C10	176.0 (4)	C17—C18—C21—F9B	−63.1 (12)
O2—C8—C13—C12	−176.8 (4)	C18—C19—C20—C15	0.7 (5)
O3—P2—O4—C22	−178.3 (2)	C19—C18—C21—F7A	177.9 (14)
O3—P2—N1—P1	−99.69 (16)	C19—C18—C21—F8A	−57.2 (10)
O3—P2—N2—P3	102.25 (17)	C19—C18—C21—F9A	53.2 (11)
O3—C15—C16—C17	−174.9 (2)	C19—C18—C21—F8B	−11.4 (15)
O3—C15—C20—C19	174.7 (3)	C19—C18—C21—F7B	−133.0 (9)
O4—P2—O3—C15	79.2 (2)	C19—C18—C21—F9B	115.4 (11)
O4—P2—N1—P1	150.91 (14)	C20—C15—C16—C17	1.3 (4)
O4—P2—N2—P3	−150.68 (15)	C21—C18—C19—C20	−177.8 (3)
O4—C22—C23—C24	−175.1 (3)	C22—C23—C24—C25	−0.6 (7)
O4—C22—C27—C26	175.4 (4)	C23—C22—C27—C26	0.1 (6)
O5—P3—O6—C36	179.9 (3)	C23—C24—C25—C26	0.5 (7)
O5—P3—N2—P2	−111.19 (18)	C23—C24—C25—C28	−179.9 (5)
O5—P3—N3—P1	116.65 (19)	C24—C25—C26—C27	−0.1 (8)
O5—C29—C30—C31	−177.0 (3)	C24—C25—C28—F10A	106.2 (14)
O5—C29—C34—C33	176.8 (3)	C24—C25—C28—F11A	−128.8 (9)
O6—P3—O5—C29	175.6 (3)	C24—C25—C28—F12A	−13.4 (14)
O6—P3—N2—P2	145.06 (16)	C24—C25—C28—F10B	69 (2)
O6—P3—N3—P1	−139.50 (18)	C24—C25—C28—F12B	−43 (2)
O6—C36—C37—C38	−176.9 (4)	C24—C25—C28—F11B	−170.1 (18)
O6—C36—C41—C40	176.3 (3)	C25—C26—C27—C22	−0.2 (8)
N1—P1—O1—C1	48.4 (3)	C26—C25—C28—F10A	−74.2 (15)
N1—P1—O2—C8	47.0 (3)	C26—C25—C28—F11A	50.8 (10)
N1—P1—N3—P3	12.4 (2)	C26—C25—C28—F12A	166.2 (12)
N1—P2—O3—C15	−36.7 (2)	C26—C25—C28—F10B	−111 (2)
N1—P2—O4—C22	−60.9 (2)	C26—C25—C28—F12B	137 (2)
N1—P2—N2—P3	−23.5 (2)	C26—C25—C28—F11B	9.5 (19)
N2—P2—O3—C15	−165.6 (2)	C27—C22—C23—C24	0.3 (6)
N2—P2—O4—C22	70.2 (2)	C28—C25—C26—C27	−179.7 (5)
N2—P2—N1—P1	23.3 (2)	C29—C30—C31—C32	0.5 (6)
N2—P3—O5—C29	61.3 (3)	C30—C29—C34—C33	−0.2 (6)
N2—P3—O6—C36	−65.3 (3)	C30—C31—C32—C33	−0.8 (6)
N2—P3—N3—P1	−12.3 (2)	C30—C31—C32—C35	−179.6 (4)
N3—P1—O1—C1	177.2 (2)	C31—C32—C33—C34	0.5 (6)
N3—P1—O2—C8	−84.4 (3)	C31—C32—C35—F13A	77.2 (15)
N3—P1—N1—P2	−17.8 (2)	C31—C32—C35—F14A	−40.8 (17)
N3—P3—O5—C29	−70.0 (3)	C31—C32—C35—F15A	−163.1 (18)
N3—P3—O6—C36	64.8 (3)	C31—C32—C35—F14B	3.0 (13)
N3—P3—N2—P2	17.8 (2)	C31—C32—C35—F15B	−121.1 (11)
C1—C2—C3—C4	0.5 (5)	C31—C32—C35—F13B	122.5 (12)
C2—C1—C6—C5	−0.3 (5)	C32—C33—C34—C29	0.0 (6)
C2—C3—C4—C5	0.0 (5)	C33—C32—C35—F13A	−101.7 (15)
C2—C3—C4—C7	177.9 (4)	C33—C32—C35—F14A	140.4 (16)
C3—C4—C5—C6	−0.8 (5)	C33—C32—C35—F15A	18.1 (18)
C3—C4—C7—F1A	5.8 (12)	C33—C32—C35—F14B	−175.8 (12)
C3—C4—C7—F2A	−109.5 (7)	C33—C32—C35—F15B	60.1 (12)
C3—C4—C7—F3A	128.1 (15)	C33—C32—C35—F13B	−56.3 (13)
C3—C4—C7—F1B	−76.6 (17)	C34—C29—C30—C31	0.0 (6)
C3—C4—C7—F3B	50.0 (17)	C35—C32—C33—C34	179.4 (4)
C3—C4—C7—F2B	168.0 (10)	C36—C37—C38—C39	2.2 (8)
C4—C5—C6—C1	0.9 (5)	C37—C36—C41—C40	2.1 (6)
C5—C4—C7—F1A	−176.4 (10)	C37—C38—C39—C40	−1.0 (7)
C5—C4—C7—F2A	68.3 (8)	C37—C38—C39—C42	178.8 (5)
C5—C4—C7—F3A	−54.1 (15)	C38—C39—C40—C41	0.4 (7)
C5—C4—C7—F1B	101.2 (17)	C38—C39—C42—F16A	168.6 (12)
C5—C4—C7—F3B	−132.1 (16)	C38—C39—C42—F17A	49.1 (10)
C5—C4—C7—F2B	−14.2 (11)	C38—C39—C42—F18A	−65.9 (13)
C6—C1—C2—C3	−0.4 (5)	C38—C39—C42—F18B	−7.6 (18)
C7—C4—C5—C6	−178.6 (4)	C38—C39—C42—F16B	−129.5 (8)
C8—C9—C10—C11	−0.5 (9)	C38—C39—C42—F17B	116.2 (16)
C9—C8—C13—C12	−1.8 (7)	C39—C40—C41—C36	−1.0 (7)
C9—C10—C11—C12	0.0 (8)	C40—C39—C42—F16A	−11.6 (14)
C9—C10—C11—C14	−176.6 (5)	C40—C39—C42—F17A	−131.1 (8)
C10—C11—C12—C13	−0.5 (7)	C40—C39—C42—F18A	113.8 (12)
C10—C11—C14—F4A	−100.9 (16)	C40—C39—C42—F18B	172.2 (17)
C10—C11—C14—F5A	141.4 (16)	C40—C39—C42—F16B	50.2 (9)
C10—C11—C14—F6A	16.3 (17)	C40—C39—C42—F17B	−64.1 (16)
C10—C11—C14—F5B	113.7 (17)	C41—C36—C37—C38	−2.7 (7)
C10—C11—C14—F4B	−126.6 (17)	C42—C39—C40—C41	−179.4 (4)

### Hexakis[4-(trifluoromethyl)phenoxy]cyclotriphosphazene (2) Hydrogen-bond geometry (Å, º)

**Table:**

*D*—H···*A*	*D*—H	H···*A*	*D*···*A*	*D*—H···*A*
C13—H13···F7*A*^i^	0.93	2.64	3.475 (14)	149
C13—H13···F7*B*^i^	0.93	2.52	3.316 (11)	144
C37—H37···F7*A*^ii^	0.93	2.33	3.149 (8)	146

Symmetry codes: (i) −*x*+1, −*y*+1, −*z*+1; (ii) *x*, *y*+1, *z*.

### Hexakis[3,5-bis(trifluoromethyl)phenoxy]cyclotriphosphazene (3) Crystal data

**Table:**

C_48_H_18_F_36_N_3_O_6_P_3_	*Z* = 2
*M**_r_* = 1509.56	*F*(000) = 1488
Triclinic, *P*1	*D*_x_ = 1.743 Mg m^−^^3^
*a* = 8.9782 (14) Å	Mo *K*α radiation, λ = 0.71073 Å
*b* = 13.947 (2) Å	Cell parameters from 4291 reflections
*c* = 23.205 (4) Å	θ = 2.3–28.0°
α = 97.276 (6)°	µ = 0.27 mm^−^^1^
β = 93.155 (6)°	*T* = 298 K
γ = 91.615 (6)°	Needle, colorless
*V* = 2876.2 (8) Å^3^	0.18 × 0.09 × 0.08 mm

### Hexakis[3,5-bis(trifluoromethyl)phenoxy]cyclotriphosphazene (3) Data collection

**Table:**

Bruker SMART CCD area detector diffractometer	6133 reflections with *I* > 2σ(*I*)
Graphite monochromator	*R*_int_ = 0.046
phi and ω scans	θ_max_ = 28.7°, θ_min_ = 1.8°
Absorption correction: multi-scan (SADABS; Bruker, 2001)	*h* = −12→11
*T*_min_ = 0.629, *T*_max_ = 0.9	*k* = −18→18
28557 measured reflections	*l* = −26→31
14187 independent reflections	

### Hexakis[3,5-bis(trifluoromethyl)phenoxy]cyclotriphosphazene (3) Refinement

**Table:**

Refinement on *F*^2^	813 restraints
Least-squares matrix: full	Hydrogen site location: inferred from neighbouring sites
*R*[*F*^2^ > 2σ(*F*^2^)] = 0.063	H-atom parameters constrained
*wR*(*F*^2^) = 0.160	*w* = 1/[σ^2^(*F*_o_^2^) + (0.0608*P*)^2^] where *P* = (*F*_o_^2^ + 2*F*_c_^2^)/3
*S* = 0.95	(Δ/σ)_max_ = 0.001
14187 reflections	Δρ_max_ = 0.28 e Å^−^^3^
1201 parameters	Δρ_min_ = −0.32 e Å^−^^3^

### Hexakis[3,5-bis(trifluoromethyl)phenoxy]cyclotriphosphazene (3) Special details

**Table:**

Experimental. The data collection nominally covered a full sphere of reciprocal space by a combination of 4 sets of ω scans each set at different φ and/or 2θ angles and each scan (30 s exposure) covering -0.300° degrees in ω. The crystal to detector distance was 5.82 cm.
Geometry. All esds (except the esd in the dihedral angle between two l.s. planes) are estimated using the full covariance matrix. The cell esds are taken into account individually in the estimation of esds in distances, angles and torsion angles; correlations between esds in cell parameters are only used when they are defined by crystal symmetry. An approximate (isotropic) treatment of cell esds is used for estimating esds involving l.s. planes.

### Hexakis[3,5-bis(trifluoromethyl)phenoxy]cyclotriphosphazene (3) Fractional atomic coordinates and isotropic or equivalent isotropic displacement parameters (Å^2^)

**Table:**

	*x*	*y*	*z*	*U*_iso_*/*U*_eq_	Occ. (<1)
P1	0.71031 (8)	0.68279 (5)	0.24145 (3)	0.0490 (2)	
P2	0.94354 (8)	0.55920 (6)	0.22697 (3)	0.0517 (2)	
P3	0.83033 (8)	0.64836 (6)	0.13597 (3)	0.0502 (2)	
F1	0.5896 (14)	0.3149 (7)	0.1837 (4)	0.141 (3)	0.70 (2)
F1A	0.638 (2)	0.2998 (19)	0.2025 (15)	0.168 (11)	0.30 (2)
F2	0.3883 (10)	0.2580 (10)	0.2101 (7)	0.176 (5)	0.70 (2)
F2A	0.407 (3)	0.2829 (19)	0.1866 (10)	0.125 (7)	0.30 (2)
F3	0.5878 (17)	0.2466 (8)	0.2585 (4)	0.146 (4)	0.70 (2)
F3A	0.509 (5)	0.2257 (13)	0.2538 (10)	0.168 (11)	0.30 (2)
F4	0.3888 (19)	0.5530 (13)	0.4454 (5)	0.148 (6)	0.56 (2)
F4A	0.4169 (18)	0.5037 (18)	0.4526 (6)	0.137 (6)	0.44 (2)
F5	0.335 (2)	0.4079 (10)	0.4299 (8)	0.186 (6)	0.56 (2)
F5A	0.265 (2)	0.4013 (10)	0.4116 (7)	0.142 (6)	0.44 (2)
F6	0.1917 (8)	0.5108 (11)	0.3996 (6)	0.126 (4)	0.56 (2)
F6A	0.229 (3)	0.5491 (16)	0.4143 (9)	0.178 (8)	0.44 (2)
F7	0.769 (3)	0.6724 (9)	0.4646 (8)	0.175 (9)	0.39 (2)
F7A	0.8440 (14)	0.7015 (10)	0.4699 (5)	0.158 (5)	0.61 (2)
F8	0.625 (2)	0.7553 (14)	0.5101 (8)	0.173 (7)	0.39 (2)
F8A	0.6167 (12)	0.7081 (11)	0.4797 (5)	0.181 (6)	0.61 (2)
F9	0.850 (2)	0.8013 (16)	0.5129 (8)	0.210 (9)	0.39 (2)
F9A	0.7620 (19)	0.8100 (6)	0.5286 (3)	0.166 (5)	0.61 (2)
F10	0.7428 (13)	1.1287 (8)	0.4067 (9)	0.221 (9)	0.506 (16)
F10A	0.736 (2)	1.1168 (8)	0.4408 (4)	0.203 (7)	0.494 (16)
F11	0.532 (2)	1.0946 (8)	0.3626 (4)	0.179 (7)	0.506 (16)
F11A	0.6613 (16)	1.1202 (5)	0.3594 (3)	0.117 (4)	0.494 (16)
F12	0.5696 (12)	1.0901 (7)	0.4511 (3)	0.119 (4)	0.506 (16)
F12A	0.5012 (15)	1.0878 (9)	0.4176 (11)	0.249 (11)	0.494 (16)
F13	1.144 (2)	0.1360 (10)	0.2463 (5)	0.150 (5)	0.63 (3)
F13A	1.079 (4)	0.108 (2)	0.2450 (8)	0.172 (9)	0.37 (3)
F14	1.2099 (16)	0.1545 (10)	0.3339 (7)	0.171 (5)	0.63 (3)
F14A	1.2408 (14)	0.1559 (16)	0.3070 (16)	0.159 (9)	0.37 (3)
F15	1.0045 (16)	0.0813 (9)	0.3040 (9)	0.165 (5)	0.63 (3)
F15A	1.054 (4)	0.092 (2)	0.3328 (16)	0.207 (12)	0.37 (3)
F16	0.7576 (18)	0.4766 (8)	0.4282 (6)	0.122 (5)	0.46 (2)
F16A	0.860 (3)	0.4824 (7)	0.4431 (6)	0.177 (7)	0.54 (2)
F17	0.7121 (18)	0.3375 (7)	0.4346 (8)	0.160 (7)	0.46 (2)
F17A	0.6836 (9)	0.3799 (16)	0.4191 (5)	0.179 (6)	0.54 (2)
F18	0.9175 (16)	0.400 (2)	0.4686 (6)	0.191 (10)	0.46 (2)
F18A	0.8660 (18)	0.3414 (10)	0.4653 (6)	0.143 (5)	0.54 (2)
F19	1.206 (3)	0.8001 (8)	0.4410 (5)	0.241 (11)	0.512 (18)
F19A	1.1026 (12)	0.8208 (11)	0.4371 (5)	0.142 (5)	0.488 (18)
F20	1.2892 (11)	0.9298 (9)	0.4176 (6)	0.155 (6)	0.512 (18)
F20A	1.3240 (10)	0.855 (2)	0.4299 (5)	0.227 (10)	0.488 (18)
F21	1.0671 (9)	0.9070 (11)	0.4103 (6)	0.175 (6)	0.512 (18)
F21A	1.158 (3)	0.9509 (7)	0.4111 (5)	0.257 (12)	0.488 (18)
F22	1.3408 (16)	0.9709 (5)	0.2188 (6)	0.164 (5)	0.59 (2)
F22A	1.418 (2)	0.9417 (18)	0.2187 (7)	0.215 (10)	0.41 (2)
F23	1.2273 (14)	0.8765 (12)	0.1532 (5)	0.163 (6)	0.59 (2)
F23A	1.2031 (17)	0.9247 (15)	0.1795 (9)	0.151 (6)	0.41 (2)
F24	1.4405 (12)	0.8476 (8)	0.1828 (7)	0.144 (5)	0.59 (2)
F24A	1.359 (3)	0.8244 (9)	0.1555 (7)	0.161 (7)	0.41 (2)
F25	0.8228 (17)	1.1257 (11)	0.1661 (9)	0.128 (4)	0.53 (4)
F25A	0.794 (3)	1.1291 (10)	0.1456 (14)	0.160 (7)	0.47 (4)
F26	0.704 (2)	1.0263 (14)	0.2078 (5)	0.126 (4)	0.53 (4)
F26A	0.749 (4)	1.042 (2)	0.2111 (5)	0.178 (8)	0.47 (4)
F27	0.5995 (15)	1.0915 (14)	0.1404 (7)	0.153 (5)	0.53 (4)
F27A	0.5814 (14)	1.063 (2)	0.1454 (9)	0.183 (7)	0.47 (4)
F28	0.9200 (12)	0.8697 (11)	−0.0707 (4)	0.149 (6)	0.511 (18)
F28A	0.8986 (16)	0.9588 (14)	−0.0640 (5)	0.182 (9)	0.489 (18)
F29	0.6930 (13)	0.8624 (10)	−0.0749 (5)	0.139 (6)	0.511 (18)
F29A	0.779 (2)	0.8274 (7)	−0.0769 (5)	0.174 (9)	0.489 (18)
F30	0.810 (2)	0.9971 (6)	−0.0561 (5)	0.151 (7)	0.511 (18)
F30A	0.6690 (13)	0.9550 (13)	−0.0593 (4)	0.174 (7)	0.489 (18)
F31	0.677 (3)	0.2591 (9)	−0.0441 (4)	0.223 (9)	0.589 (19)
F31A	0.5694 (15)	0.2066 (15)	−0.0181 (11)	0.201 (11)	0.411 (19)
F32	0.5894 (11)	0.1864 (6)	0.0201 (7)	0.154 (5)	0.589 (19)
F32A	0.741 (3)	0.2175 (16)	0.0505 (7)	0.248 (13)	0.411 (19)
F33	0.8008 (9)	0.2387 (9)	0.0304 (7)	0.178 (6)	0.589 (19)
F33A	0.7772 (17)	0.2708 (11)	−0.0240 (9)	0.147 (7)	0.411 (19)
F34	0.1976 (12)	0.5304 (10)	0.0747 (5)	0.160 (5)	0.590 (17)
F34A	0.2342 (16)	0.5494 (7)	0.1064 (11)	0.182 (8)	0.410 (17)
F35	0.1807 (12)	0.3807 (9)	0.0783 (7)	0.180 (6)	0.590 (17)
F35A	0.1711 (12)	0.4226 (12)	0.0543 (4)	0.105 (4)	0.410 (17)
F36	0.2538 (11)	0.4737 (9)	0.1496 (3)	0.105 (3)	0.590 (17)
F36A	0.2470 (19)	0.4163 (18)	0.1385 (7)	0.166 (8)	0.410 (17)
O1	0.5523 (2)	0.65761 (14)	0.26291 (9)	0.0599 (5)	
O2	0.7343 (2)	0.79123 (13)	0.26994 (8)	0.0593 (5)	
O3	0.9316 (2)	0.44677 (14)	0.22645 (9)	0.0630 (6)	
O4	1.1042 (2)	0.58163 (15)	0.25806 (9)	0.0615 (6)	
O5	0.9294 (2)	0.73185 (14)	0.11666 (9)	0.0594 (5)	
O6	0.7589 (2)	0.60240 (14)	0.07457 (8)	0.0564 (5)	
N1	0.8278 (2)	0.61481 (16)	0.26621 (10)	0.0498 (6)	
N2	0.9391 (2)	0.57664 (16)	0.16180 (10)	0.0530 (6)	
N3	0.7012 (2)	0.68738 (16)	0.17451 (9)	0.0483 (6)	
C1	0.5120 (3)	0.5684 (2)	0.28023 (13)	0.0520 (7)	
C2	0.5309 (3)	0.4829 (2)	0.24578 (14)	0.0604 (8)	
H2	0.571772	0.482819	0.209819	0.073*	
C3	0.4890 (4)	0.3971 (3)	0.26466 (15)	0.0666 (9)	
C4	0.4240 (4)	0.3976 (3)	0.31689 (16)	0.0736 (9)	
H4	0.394501	0.339470	0.329313	0.088*	
C5	0.4025 (3)	0.4834 (3)	0.35074 (14)	0.0656 (9)	
C6	0.4492 (3)	0.5697 (3)	0.33279 (13)	0.0599 (8)	
H6	0.437997	0.628030	0.356175	0.072*	
C7	0.5120 (6)	0.3048 (3)	0.2289 (2)	0.0945 (12)	
C8	0.3304 (5)	0.4854 (4)	0.40642 (18)	0.0877 (12)	
C9	0.7181 (3)	0.8283 (2)	0.32754 (13)	0.0539 (7)	
C10	0.6842 (3)	0.9243 (2)	0.33655 (14)	0.0625 (8)	
H10	0.671883	0.959472	0.305258	0.075*	
C11	0.6685 (4)	0.9678 (2)	0.39261 (15)	0.0683 (9)	
C12	0.6876 (4)	0.9149 (3)	0.43872 (16)	0.0768 (10)	
H12	0.679169	0.944484	0.476579	0.092*	
C13	0.7190 (4)	0.8187 (3)	0.42867 (14)	0.0703 (9)	
C14	0.7355 (4)	0.7751 (2)	0.37316 (14)	0.0670 (9)	
H14	0.758267	0.710149	0.366485	0.080*	
C15	0.7365 (7)	0.7616 (4)	0.4775 (2)	0.1054 (14)	
C16	0.6333 (7)	1.0707 (3)	0.4025 (2)	0.1070 (14)	
C17	0.9425 (3)	0.3935 (2)	0.27387 (15)	0.0597 (8)	
C18	0.8825 (4)	0.4220 (2)	0.32527 (15)	0.0645 (9)	
H18	0.835289	0.480627	0.331163	0.077*	
C19	0.8917 (4)	0.3632 (2)	0.36890 (16)	0.0687 (9)	
C20	0.9628 (4)	0.2765 (3)	0.35954 (19)	0.0785 (10)	
H20	0.970921	0.237226	0.389009	0.094*	
C21	1.0212 (4)	0.2481 (2)	0.30702 (19)	0.0749 (10)	
C22	1.0130 (3)	0.3065 (2)	0.26336 (16)	0.0697 (9)	
H22	1.053664	0.287975	0.227808	0.084*	
C23	1.0937 (6)	0.1547 (3)	0.2982 (3)	0.1132 (16)	
C24	0.8255 (6)	0.3921 (4)	0.4240 (2)	0.0940 (12)	
C25	1.1517 (3)	0.6790 (2)	0.27201 (15)	0.0596 (8)	
C26	1.2059 (3)	0.7289 (3)	0.22998 (16)	0.0650 (8)	
H26	1.209354	0.699181	0.191890	0.078*	
C27	1.2555 (3)	0.8244 (3)	0.24508 (18)	0.0721 (9)	
C28	1.2502 (4)	0.8671 (3)	0.30172 (19)	0.0819 (10)	
H28	1.284353	0.930860	0.311921	0.098*	
C29	1.1948 (4)	0.8161 (3)	0.34337 (17)	0.0784 (10)	
C30	1.1450 (3)	0.7209 (3)	0.32856 (15)	0.0685 (9)	
H30	1.107611	0.685939	0.356459	0.082*	
C31	1.1911 (7)	0.8615 (4)	0.4038 (2)	0.1192 (16)	
C32	1.3142 (6)	0.8783 (4)	0.2013 (2)	0.1004 (13)	
C33	0.8686 (3)	0.8139 (2)	0.09617 (14)	0.0542 (8)	
C34	0.8614 (3)	0.8186 (2)	0.03791 (14)	0.0608 (8)	
H34	0.892538	0.767556	0.012114	0.073*	
C35	0.8066 (4)	0.9013 (3)	0.01763 (16)	0.0706 (9)	
C36	0.7605 (4)	0.9759 (3)	0.05658 (18)	0.0770 (10)	
H36	0.722700	1.030817	0.043019	0.092*	
C37	0.7700 (4)	0.9698 (2)	0.11528 (17)	0.0705 (9)	
C38	0.8253 (3)	0.8876 (2)	0.13500 (15)	0.0663 (9)	
H38	0.832797	0.882814	0.174652	0.080*	
C39	0.7238 (6)	1.0510 (3)	0.1561 (2)	0.0986 (13)	
C40	0.7993 (6)	0.9099 (4)	−0.04454 (19)	0.0989 (13)	
C41	0.6628 (3)	0.5212 (2)	0.07004 (12)	0.0507 (7)	
C42	0.7146 (4)	0.4337 (2)	0.04778 (13)	0.0607 (8)	
H42	0.813912	0.428079	0.038836	0.073*	
C43	0.6176 (4)	0.3537 (2)	0.03872 (14)	0.0671 (9)	
C44	0.4716 (4)	0.3614 (3)	0.05342 (14)	0.0701 (9)	
H44	0.407081	0.307288	0.047664	0.084*	
C45	0.4215 (3)	0.4495 (3)	0.07666 (13)	0.0612 (8)	
C46	0.5169 (3)	0.5302 (2)	0.08454 (12)	0.0584 (8)	
H46	0.482786	0.590061	0.099523	0.070*	
C47	0.2673 (4)	0.4588 (3)	0.09387 (19)	0.0806 (10)	
C48	0.6720 (6)	0.2609 (3)	0.0117 (3)	0.1092 (15)	

### Hexakis[3,5-bis(trifluoromethyl)phenoxy]cyclotriphosphazene (3) Atomic displacement parameters (Å^2^)

**Table:**

	*U*^11^	*U*^22^	*U*^33^	*U*^12^	*U*^13^	*U*^23^
P1	0.0555 (5)	0.0469 (5)	0.0455 (5)	0.0113 (4)	0.0045 (4)	0.0066 (4)
P2	0.0565 (5)	0.0482 (5)	0.0515 (5)	0.0133 (4)	0.0008 (4)	0.0089 (4)
P3	0.0565 (5)	0.0504 (5)	0.0445 (5)	0.0086 (4)	0.0028 (4)	0.0078 (4)
F1	0.248 (9)	0.081 (4)	0.100 (4)	0.036 (5)	0.071 (5)	0.006 (3)
F1A	0.123 (10)	0.078 (13)	0.29 (3)	−0.009 (8)	0.059 (12)	−0.054 (14)
F2	0.177 (5)	0.128 (7)	0.198 (11)	−0.052 (4)	−0.004 (5)	−0.060 (7)
F2A	0.162 (13)	0.114 (12)	0.085 (10)	0.016 (10)	−0.034 (9)	−0.018 (7)
F3	0.233 (9)	0.068 (5)	0.133 (5)	0.041 (5)	−0.017 (5)	0.005 (4)
F3A	0.33 (3)	0.067 (6)	0.103 (10)	−0.013 (13)	−0.032 (14)	0.021 (6)
F4	0.160 (10)	0.228 (11)	0.051 (5)	−0.050 (9)	0.027 (5)	0.007 (6)
F4A	0.122 (6)	0.235 (16)	0.058 (4)	−0.008 (9)	−0.008 (4)	0.043 (8)
F5	0.302 (18)	0.173 (9)	0.115 (11)	0.070 (9)	0.084 (10)	0.098 (8)
F5A	0.177 (11)	0.167 (8)	0.088 (8)	−0.068 (8)	0.040 (6)	0.046 (6)
F6	0.068 (4)	0.222 (9)	0.097 (6)	0.000 (4)	0.034 (3)	0.040 (5)
F6A	0.211 (14)	0.240 (15)	0.114 (12)	0.140 (13)	0.084 (11)	0.084 (12)
F7	0.35 (2)	0.104 (7)	0.104 (10)	0.105 (11)	0.122 (13)	0.060 (5)
F7A	0.217 (9)	0.167 (8)	0.104 (6)	0.100 (7)	−0.004 (6)	0.059 (5)
F8	0.256 (13)	0.213 (15)	0.084 (12)	0.132 (11)	0.103 (10)	0.082 (10)
F8A	0.237 (8)	0.235 (12)	0.085 (7)	−0.056 (8)	0.006 (5)	0.094 (8)
F9	0.270 (16)	0.292 (16)	0.064 (10)	0.041 (13)	−0.064 (11)	0.038 (10)
F9A	0.315 (14)	0.138 (7)	0.046 (3)	0.045 (7)	0.000 (6)	0.012 (3)
F10	0.221 (11)	0.083 (5)	0.37 (3)	−0.033 (6)	0.113 (10)	0.023 (13)
F10A	0.436 (18)	0.067 (5)	0.091 (6)	−0.054 (7)	−0.037 (8)	−0.008 (4)
F11	0.311 (18)	0.092 (7)	0.127 (7)	0.100 (9)	−0.036 (9)	−0.006 (5)
F11A	0.207 (10)	0.053 (4)	0.092 (5)	0.026 (5)	0.013 (5)	0.002 (3)
F12	0.195 (9)	0.073 (5)	0.091 (5)	0.058 (5)	0.043 (4)	−0.013 (4)
F12A	0.258 (11)	0.124 (9)	0.41 (3)	0.115 (9)	0.193 (16)	0.108 (15)
F13	0.204 (12)	0.084 (7)	0.176 (7)	0.076 (7)	0.060 (7)	0.030 (5)
F13A	0.22 (2)	0.077 (13)	0.207 (11)	0.052 (12)	−0.046 (12)	−0.025 (10)
F14	0.169 (7)	0.164 (7)	0.187 (9)	0.117 (6)	−0.011 (6)	0.036 (6)
F14A	0.106 (6)	0.122 (9)	0.24 (2)	0.051 (7)	0.011 (8)	−0.006 (12)
F15	0.202 (8)	0.057 (4)	0.249 (12)	0.023 (4)	0.048 (7)	0.046 (6)
F15A	0.24 (2)	0.109 (14)	0.32 (2)	0.097 (12)	0.14 (2)	0.131 (17)
F16	0.177 (11)	0.087 (6)	0.115 (7)	0.044 (6)	0.068 (8)	0.036 (5)
F16A	0.286 (18)	0.123 (6)	0.124 (9)	−0.003 (8)	0.087 (12)	−0.013 (5)
F17	0.234 (13)	0.093 (6)	0.164 (15)	−0.023 (7)	0.120 (11)	0.013 (6)
F17A	0.101 (5)	0.342 (19)	0.107 (5)	0.046 (7)	0.023 (4)	0.070 (10)
F18	0.171 (10)	0.33 (3)	0.064 (5)	0.072 (13)	−0.012 (6)	0.001 (12)
F18A	0.188 (10)	0.179 (9)	0.081 (6)	0.061 (7)	0.014 (6)	0.072 (7)
F19	0.48 (3)	0.170 (8)	0.071 (5)	−0.005 (15)	0.032 (15)	−0.001 (5)
F19A	0.159 (8)	0.159 (11)	0.097 (6)	−0.026 (6)	0.032 (5)	−0.030 (6)
F20	0.132 (7)	0.171 (11)	0.133 (9)	−0.046 (6)	−0.015 (6)	−0.070 (7)
F20A	0.141 (6)	0.46 (3)	0.065 (5)	−0.047 (10)	−0.028 (5)	0.016 (12)
F21	0.137 (6)	0.205 (13)	0.154 (8)	−0.022 (6)	0.043 (5)	−0.095 (9)
F21A	0.52 (3)	0.108 (7)	0.132 (8)	0.014 (14)	0.01 (2)	−0.045 (6)
F22	0.193 (10)	0.070 (4)	0.237 (10)	−0.011 (5)	0.097 (7)	0.023 (4)
F22A	0.202 (15)	0.30 (2)	0.144 (12)	−0.178 (15)	−0.024 (11)	0.073 (12)
F23	0.137 (7)	0.232 (14)	0.138 (8)	−0.032 (7)	−0.006 (6)	0.107 (8)
F23A	0.167 (9)	0.165 (13)	0.141 (12)	0.024 (7)	0.011 (8)	0.094 (10)
F24	0.111 (6)	0.156 (9)	0.187 (11)	0.042 (6)	0.070 (6)	0.070 (7)
F24A	0.178 (16)	0.199 (9)	0.119 (10)	0.001 (10)	0.070 (11)	0.040 (6)
F25	0.159 (8)	0.096 (6)	0.116 (9)	−0.026 (4)	0.022 (5)	−0.032 (5)
F25A	0.281 (16)	0.052 (5)	0.148 (15)	−0.006 (6)	0.059 (11)	−0.006 (6)
F26	0.184 (9)	0.096 (9)	0.108 (5)	0.042 (5)	0.068 (5)	0.017 (6)
F26A	0.36 (2)	0.080 (7)	0.093 (5)	0.037 (12)	0.029 (8)	−0.013 (5)
F27	0.164 (7)	0.110 (8)	0.169 (9)	0.069 (6)	−0.046 (9)	−0.032 (6)
F27A	0.151 (7)	0.170 (16)	0.237 (13)	0.083 (8)	0.073 (8)	0.012 (10)
F28	0.173 (7)	0.200 (14)	0.090 (5)	0.019 (8)	0.043 (5)	0.064 (7)
F28A	0.185 (11)	0.27 (2)	0.098 (7)	−0.132 (13)	−0.003 (8)	0.061 (13)
F29	0.158 (8)	0.160 (12)	0.093 (5)	−0.053 (7)	−0.053 (6)	0.031 (8)
F29A	0.33 (3)	0.120 (6)	0.066 (4)	−0.047 (8)	−0.008 (11)	0.020 (4)
F30	0.27 (2)	0.100 (5)	0.095 (5)	−0.019 (6)	0.022 (9)	0.060 (4)
F30A	0.177 (9)	0.245 (17)	0.111 (6)	0.033 (10)	−0.038 (5)	0.077 (8)
F31	0.43 (3)	0.110 (9)	0.131 (5)	0.090 (15)	0.093 (11)	−0.020 (5)
F31A	0.176 (9)	0.109 (14)	0.29 (3)	−0.036 (9)	0.078 (10)	−0.121 (14)
F32	0.148 (6)	0.051 (3)	0.266 (15)	0.020 (3)	0.033 (7)	0.017 (6)
F32A	0.40 (3)	0.135 (13)	0.229 (13)	0.159 (19)	0.035 (16)	0.070 (10)
F33	0.113 (5)	0.106 (7)	0.311 (16)	0.050 (4)	0.044 (6)	−0.014 (8)
F33A	0.149 (10)	0.080 (7)	0.211 (16)	0.024 (6)	0.096 (10)	−0.025 (9)
F34	0.102 (6)	0.267 (11)	0.144 (7)	0.090 (8)	0.053 (5)	0.126 (8)
F34A	0.109 (9)	0.112 (7)	0.31 (2)	0.004 (6)	0.110 (13)	−0.052 (9)
F35	0.104 (6)	0.200 (8)	0.212 (14)	−0.055 (6)	0.056 (7)	−0.080 (8)
F35A	0.053 (4)	0.180 (11)	0.078 (5)	−0.005 (6)	−0.009 (3)	0.010 (5)
F36	0.081 (3)	0.177 (8)	0.059 (3)	0.037 (5)	0.020 (2)	0.014 (3)
F36A	0.121 (7)	0.31 (2)	0.094 (9)	0.015 (13)	0.047 (7)	0.089 (12)
O1	0.0539 (12)	0.0624 (13)	0.0681 (14)	0.0181 (10)	0.0136 (10)	0.0192 (11)
O2	0.0897 (15)	0.0476 (12)	0.0408 (12)	0.0092 (10)	0.0109 (10)	0.0017 (9)
O3	0.0852 (15)	0.0487 (13)	0.0559 (14)	0.0142 (10)	0.0007 (11)	0.0089 (10)
O4	0.0564 (12)	0.0587 (14)	0.0696 (15)	0.0184 (10)	−0.0073 (10)	0.0105 (11)
O5	0.0577 (12)	0.0558 (13)	0.0669 (14)	0.0059 (10)	0.0024 (10)	0.0160 (11)
O6	0.0687 (13)	0.0608 (13)	0.0396 (12)	0.0018 (10)	0.0028 (10)	0.0065 (10)
N1	0.0526 (13)	0.0511 (14)	0.0470 (15)	0.0135 (11)	0.0007 (11)	0.0098 (11)
N2	0.0624 (15)	0.0535 (15)	0.0448 (15)	0.0195 (12)	0.0064 (11)	0.0077 (12)
N3	0.0514 (13)	0.0556 (15)	0.0389 (14)	0.0149 (11)	0.0014 (10)	0.0072 (11)
C1	0.0498 (16)	0.065 (2)	0.0440 (18)	0.0066 (15)	0.0028 (14)	0.0183 (16)
C2	0.0651 (19)	0.068 (2)	0.049 (2)	0.0057 (17)	0.0020 (15)	0.0092 (17)
C3	0.076 (2)	0.064 (2)	0.060 (2)	−0.0032 (17)	−0.0021 (17)	0.0126 (17)
C4	0.077 (2)	0.077 (2)	0.069 (2)	−0.0079 (19)	−0.0033 (19)	0.025 (2)
C5	0.0576 (19)	0.092 (3)	0.051 (2)	−0.0016 (18)	0.0010 (15)	0.0239 (19)
C6	0.0504 (17)	0.080 (2)	0.050 (2)	0.0108 (16)	0.0016 (14)	0.0122 (17)
C7	0.127 (4)	0.066 (3)	0.089 (3)	−0.006 (3)	0.001 (3)	0.007 (2)
C8	0.085 (3)	0.119 (4)	0.066 (3)	0.003 (3)	0.014 (2)	0.034 (3)
C9	0.0623 (19)	0.0522 (19)	0.0470 (19)	0.0117 (15)	0.0050 (15)	0.0032 (15)
C10	0.078 (2)	0.0499 (19)	0.059 (2)	0.0026 (16)	0.0052 (17)	0.0048 (16)
C11	0.090 (2)	0.054 (2)	0.059 (2)	0.0098 (17)	0.0122 (18)	−0.0065 (17)
C12	0.097 (3)	0.075 (2)	0.055 (2)	0.012 (2)	0.0048 (19)	−0.0040 (19)
C13	0.094 (3)	0.070 (2)	0.046 (2)	0.0174 (19)	−0.0008 (18)	0.0048 (17)
C14	0.088 (2)	0.062 (2)	0.050 (2)	0.0221 (18)	0.0030 (18)	0.0026 (17)
C15	0.157 (5)	0.104 (4)	0.060 (3)	0.040 (3)	0.011 (3)	0.020 (3)
C16	0.171 (5)	0.065 (3)	0.085 (4)	0.021 (3)	0.028 (3)	−0.004 (3)
C17	0.065 (2)	0.0488 (19)	0.066 (2)	0.0052 (15)	−0.0057 (17)	0.0144 (17)
C18	0.077 (2)	0.057 (2)	0.061 (2)	0.0142 (17)	−0.0022 (18)	0.0140 (17)
C19	0.076 (2)	0.065 (2)	0.068 (2)	0.0041 (18)	−0.0016 (17)	0.0204 (18)
C20	0.075 (2)	0.067 (2)	0.099 (3)	0.0040 (19)	−0.009 (2)	0.038 (2)
C21	0.075 (2)	0.051 (2)	0.102 (3)	0.0146 (17)	0.001 (2)	0.025 (2)
C22	0.071 (2)	0.055 (2)	0.085 (3)	0.0144 (17)	0.0084 (18)	0.0122 (18)
C23	0.126 (4)	0.072 (3)	0.150 (5)	0.040 (3)	0.016 (4)	0.038 (3)
C24	0.120 (4)	0.093 (3)	0.075 (3)	0.018 (3)	0.004 (3)	0.027 (3)
C25	0.0466 (17)	0.065 (2)	0.068 (2)	0.0155 (15)	−0.0074 (16)	0.0101 (18)
C26	0.0552 (19)	0.070 (2)	0.069 (2)	0.0109 (16)	−0.0029 (16)	0.0071 (18)
C27	0.058 (2)	0.074 (2)	0.086 (3)	0.0002 (17)	0.0006 (18)	0.020 (2)
C28	0.072 (2)	0.071 (2)	0.098 (3)	−0.0002 (19)	−0.007 (2)	−0.001 (2)
C29	0.077 (2)	0.084 (3)	0.070 (2)	−0.003 (2)	−0.0074 (19)	−0.003 (2)
C30	0.063 (2)	0.082 (2)	0.060 (2)	0.0054 (18)	−0.0044 (17)	0.0103 (19)
C31	0.135 (4)	0.121 (4)	0.089 (4)	−0.021 (4)	−0.006 (3)	−0.020 (3)
C32	0.097 (3)	0.089 (3)	0.119 (4)	−0.010 (3)	0.009 (3)	0.027 (3)
C33	0.0542 (18)	0.0521 (19)	0.057 (2)	−0.0031 (15)	−0.0002 (15)	0.0144 (16)
C34	0.068 (2)	0.054 (2)	0.060 (2)	−0.0099 (15)	−0.0004 (16)	0.0106 (16)
C35	0.083 (2)	0.068 (2)	0.062 (2)	−0.0127 (19)	−0.0100 (18)	0.0238 (18)
C36	0.082 (2)	0.061 (2)	0.091 (3)	−0.0016 (18)	−0.001 (2)	0.027 (2)
C37	0.085 (2)	0.051 (2)	0.078 (3)	0.0032 (17)	0.012 (2)	0.0125 (18)
C38	0.078 (2)	0.059 (2)	0.063 (2)	0.0021 (17)	0.0086 (17)	0.0109 (17)
C39	0.130 (4)	0.065 (3)	0.103 (3)	0.018 (3)	0.019 (3)	0.012 (3)
C40	0.128 (4)	0.093 (3)	0.078 (3)	−0.022 (3)	−0.012 (3)	0.034 (3)
C41	0.0614 (18)	0.0562 (19)	0.0354 (17)	0.0058 (15)	−0.0015 (14)	0.0098 (14)
C42	0.067 (2)	0.060 (2)	0.058 (2)	0.0176 (16)	0.0119 (16)	0.0109 (16)
C43	0.086 (2)	0.0490 (19)	0.067 (2)	0.0144 (17)	0.0115 (18)	0.0068 (16)
C44	0.082 (2)	0.065 (2)	0.064 (2)	−0.0040 (18)	0.0073 (18)	0.0112 (18)
C45	0.0589 (18)	0.073 (2)	0.050 (2)	0.0050 (17)	0.0057 (15)	0.0009 (17)
C46	0.0618 (19)	0.066 (2)	0.0452 (19)	0.0126 (16)	0.0032 (15)	−0.0024 (15)
C47	0.068 (2)	0.104 (3)	0.068 (3)	−0.004 (2)	0.010 (2)	0.002 (2)
C48	0.134 (4)	0.058 (3)	0.138 (5)	0.016 (3)	0.037 (3)	0.006 (3)

### Hexakis[3,5-bis(trifluoromethyl)phenoxy]cyclotriphosphazene (3) Geometric parameters (Å, º)

**Table:**

P1—O1	1.574 (2)	F34—C47	1.304 (8)
P1—O2	1.576 (2)	F34A—C47	1.306 (10)
P1—N1	1.571 (2)	F35—C47	1.320 (8)
P1—N3	1.561 (2)	F35A—C47	1.271 (9)
P2—O3	1.567 (2)	F36—C47	1.297 (7)
P2—O4	1.582 (2)	F36A—C47	1.276 (10)
P2—N1	1.575 (2)	O1—C1	1.400 (3)
P2—N2	1.560 (2)	O2—C9	1.388 (3)
P3—O5	1.570 (2)	O3—C17	1.404 (4)
P3—O6	1.580 (2)	O4—C25	1.405 (4)
P3—N2	1.567 (2)	O5—C33	1.406 (3)
P3—N3	1.570 (2)	O6—C41	1.394 (3)
F1—C7	1.312 (8)	C1—C2	1.369 (4)
F1A—C7	1.313 (12)	C1—C6	1.370 (4)
F2—C7	1.297 (8)	C2—H2	0.9300
F2A—C7	1.322 (12)	C2—C3	1.374 (4)
F3—C7	1.309 (7)	C3—C4	1.373 (5)
F3A—C7	1.309 (12)	C3—C7	1.468 (5)
F4—C8	1.298 (9)	C4—H4	0.9300
F4A—C8	1.282 (10)	C4—C5	1.371 (5)
F5—C8	1.271 (9)	C5—C6	1.385 (4)
F5A—C8	1.319 (10)	C5—C8	1.474 (5)
F6—C8	1.311 (8)	C6—H6	0.9300
F6A—C8	1.293 (10)	C9—C10	1.374 (4)
F7—C15	1.289 (10)	C9—C14	1.372 (4)
F7A—C15	1.301 (8)	C10—H10	0.9300
F8—C15	1.299 (10)	C10—C11	1.381 (4)
F8A—C15	1.298 (8)	C11—C12	1.380 (5)
F9—C15	1.336 (11)	C11—C16	1.470 (5)
F9A—C15	1.293 (8)	C12—H12	0.9300
F10—C16	1.247 (9)	C12—C13	1.373 (4)
F10A—C16	1.335 (10)	C13—C14	1.371 (4)
F11—C16	1.341 (9)	C13—C15	1.468 (5)
F11A—C16	1.317 (8)	C14—H14	0.9300
F12—C16	1.294 (8)	C17—C18	1.354 (4)
F12A—C16	1.276 (9)	C17—C22	1.385 (4)
F13—C23	1.307 (9)	C18—H18	0.9300
F13A—C23	1.319 (13)	C18—C19	1.382 (4)
F14—C23	1.297 (9)	C19—C20	1.381 (4)
F14A—C23	1.324 (12)	C19—C24	1.456 (5)
F15—C23	1.308 (9)	C20—H20	0.9300
F15A—C23	1.311 (12)	C20—C21	1.370 (5)
F16—C24	1.337 (8)	C21—C22	1.378 (5)
F16A—C24	1.304 (8)	C21—C23	1.469 (5)
F17—C24	1.307 (10)	C22—H22	0.9300
F17A—C24	1.277 (9)	C25—C26	1.370 (4)
F18—C24	1.280 (9)	C25—C30	1.372 (4)
F18A—C24	1.303 (8)	C26—H26	0.9300
F19—C31	1.295 (9)	C26—C27	1.388 (4)
F19A—C31	1.305 (10)	C27—C28	1.377 (5)
F20—C31	1.277 (9)	C27—C32	1.451 (5)
F20A—C31	1.320 (9)	C28—H28	0.9300
F21—C31	1.304 (10)	C28—C29	1.376 (5)
F21A—C31	1.284 (10)	C29—C30	1.383 (5)
F22—C32	1.316 (8)	C29—C31	1.465 (6)
F22A—C32	1.282 (10)	C30—H30	0.9300
F23—C32	1.322 (8)	C33—C34	1.360 (4)
F23A—C32	1.316 (10)	C33—C38	1.360 (4)
F24—C32	1.298 (7)	C34—H34	0.9300
F24A—C32	1.308 (10)	C34—C35	1.392 (4)
F25—C39	1.340 (10)	C35—C36	1.378 (5)
F25A—C39	1.300 (11)	C35—C40	1.461 (5)
F26—C39	1.310 (10)	C36—H36	0.9300
F26A—C39	1.305 (11)	C36—C37	1.374 (5)
F27—C39	1.319 (10)	C37—C38	1.382 (4)
F27A—C39	1.309 (10)	C37—C39	1.467 (5)
F28—C40	1.371 (7)	C38—H38	0.9300
F28A—C40	1.245 (9)	C41—C42	1.368 (4)
F29—C40	1.272 (9)	C41—C46	1.375 (4)
F29A—C40	1.294 (9)	C42—H42	0.9300
F30—C40	1.280 (8)	C42—C43	1.383 (4)
F30A—C40	1.391 (8)	C43—C44	1.376 (4)
F31—C48	1.295 (9)	C43—C48	1.473 (5)
F31A—C48	1.288 (10)	C44—H44	0.9300
F32—C48	1.301 (8)	C44—C45	1.376 (4)
F32A—C48	1.287 (11)	C45—C46	1.382 (4)
F33—C48	1.273 (9)	C45—C47	1.467 (4)
F33A—C48	1.305 (10)	C46—H46	0.9300
			
O1—P1—O2	100.80 (11)	F15A—C23—C21	114.9 (13)
N1—P1—O1	108.95 (12)	F16—C24—C19	115.6 (6)
N1—P1—O2	112.37 (12)	F16A—C24—C19	110.7 (7)
N3—P1—O1	110.77 (11)	F17—C24—F16	98.1 (7)
N3—P1—O2	104.87 (12)	F17—C24—C19	114.9 (8)
N3—P1—N1	117.74 (12)	F17A—C24—F16A	109.6 (9)
O3—P2—O4	100.23 (11)	F17A—C24—F18A	103.8 (8)
O3—P2—N1	113.20 (12)	F17A—C24—C19	111.2 (7)
N1—P2—O4	107.77 (12)	F18—C24—F16	104.4 (10)
N2—P2—O3	105.87 (12)	F18—C24—F17	107.6 (9)
N2—P2—O4	111.95 (12)	F18—C24—C19	114.5 (8)
N2—P2—N1	116.65 (12)	F18A—C24—F16A	107.0 (9)
O5—P3—O6	99.58 (11)	F18A—C24—C19	114.2 (7)
N2—P3—O5	107.08 (12)	C26—C25—O4	120.0 (3)
N2—P3—O6	110.66 (12)	C26—C25—C30	121.8 (3)
N2—P3—N3	117.22 (12)	C30—C25—O4	118.1 (3)
N3—P3—O5	112.58 (12)	C25—C26—H26	120.5
N3—P3—O6	108.31 (11)	C25—C26—C27	119.0 (3)
C1—O1—P1	124.12 (17)	C27—C26—H26	120.5
C9—O2—P1	127.37 (19)	C26—C27—C32	119.9 (4)
C17—O3—P2	128.4 (2)	C28—C27—C26	119.7 (4)
C25—O4—P2	117.78 (16)	C28—C27—C32	120.4 (4)
C33—O5—P3	122.75 (17)	C27—C28—H28	119.7
C41—O6—P3	120.21 (17)	C29—C28—C27	120.5 (4)
P1—N1—P2	122.41 (15)	C29—C28—H28	119.7
P2—N2—P3	122.79 (15)	C28—C29—C30	120.0 (4)
P1—N3—P3	120.72 (13)	C28—C29—C31	120.3 (4)
C2—C1—O1	121.8 (3)	C30—C29—C31	119.6 (4)
C2—C1—C6	120.9 (3)	C25—C30—C29	118.9 (3)
C6—C1—O1	117.3 (3)	C25—C30—H30	120.6
C1—C2—H2	120.2	C29—C30—H30	120.6
C1—C2—C3	119.6 (3)	F19—C31—F21	110.4 (11)
C3—C2—H2	120.2	F19—C31—C29	112.8 (7)
C2—C3—C7	120.2 (4)	F19A—C31—F20A	102.8 (9)
C4—C3—C2	120.1 (3)	F19A—C31—C29	116.9 (7)
C4—C3—C7	119.6 (4)	F20—C31—F19	108.2 (10)
C3—C4—H4	119.9	F20—C31—F21	101.9 (7)
C5—C4—C3	120.1 (3)	F20—C31—C29	113.5 (8)
C5—C4—H4	119.9	F20A—C31—C29	108.3 (9)
C4—C5—C6	119.9 (3)	F21—C31—C29	109.4 (7)
C4—C5—C8	121.0 (4)	F21A—C31—F19A	104.0 (10)
C6—C5—C8	119.1 (4)	F21A—C31—F20A	107.5 (10)
C1—C6—C5	119.3 (3)	F21A—C31—C29	116.3 (8)
C1—C6—H6	120.3	F22—C32—F23	104.3 (8)
C5—C6—H6	120.3	F22—C32—C27	114.4 (7)
F1—C7—C3	112.8 (5)	F22A—C32—F23A	106.7 (12)
F1A—C7—F2A	104.4 (13)	F22A—C32—F24A	107.6 (10)
F1A—C7—C3	115.3 (10)	F22A—C32—C27	116.5 (8)
F2—C7—F1	107.7 (7)	F23—C32—C27	115.1 (6)
F2—C7—F3	106.0 (7)	F23A—C32—C27	107.5 (9)
F2—C7—C3	113.2 (7)	F24—C32—F22	104.2 (7)
F2A—C7—C3	113.0 (12)	F24—C32—F23	104.0 (7)
F3—C7—F1	105.3 (7)	F24—C32—C27	113.6 (6)
F3—C7—C3	111.2 (6)	F24A—C32—F23A	103.0 (9)
F3A—C7—F1A	103.1 (14)	F24A—C32—C27	114.4 (7)
F3A—C7—F2A	100.8 (13)	C34—C33—O5	118.6 (3)
F3A—C7—C3	118.3 (12)	C38—C33—O5	119.4 (3)
F4—C8—F6	103.2 (9)	C38—C33—C34	122.0 (3)
F4—C8—C5	111.8 (7)	C33—C34—H34	120.6
F4A—C8—F5A	104.1 (10)	C33—C34—C35	118.7 (3)
F4A—C8—F6A	103.2 (12)	C35—C34—H34	120.6
F4A—C8—C5	116.1 (9)	C34—C35—C40	120.5 (4)
F5—C8—F4	105.7 (8)	C36—C35—C34	119.7 (3)
F5—C8—F6	110.0 (9)	C36—C35—C40	119.8 (4)
F5—C8—C5	115.8 (8)	C35—C36—H36	119.7
F5A—C8—C5	111.7 (8)	C37—C36—C35	120.5 (3)
F6—C8—C5	109.5 (7)	C37—C36—H36	119.7
F6A—C8—F5A	106.2 (11)	C36—C37—C38	119.3 (3)
F6A—C8—C5	114.6 (8)	C36—C37—C39	119.7 (3)
C10—C9—O2	115.1 (3)	C38—C37—C39	121.0 (4)
C14—C9—O2	123.7 (3)	C33—C38—C37	119.7 (3)
C14—C9—C10	121.2 (3)	C33—C38—H38	120.2
C9—C10—H10	120.4	C37—C38—H38	120.2
C9—C10—C11	119.2 (3)	F25—C39—C37	114.5 (9)
C11—C10—H10	120.4	F25A—C39—F26A	108.0 (12)
C10—C11—C16	119.4 (4)	F25A—C39—F27A	106.8 (12)
C12—C11—C10	119.9 (3)	F25A—C39—C37	108.3 (11)
C12—C11—C16	120.7 (4)	F26—C39—F25	104.3 (10)
C11—C12—H12	120.0	F26—C39—F27	105.9 (10)
C13—C12—C11	120.0 (3)	F26—C39—C37	112.3 (9)
C13—C12—H12	120.0	F26A—C39—F27A	109.7 (13)
C12—C13—C15	120.2 (3)	F26A—C39—C37	115.2 (11)
C14—C13—C12	120.4 (3)	F27—C39—F25	103.3 (10)
C14—C13—C15	119.3 (3)	F27—C39—C37	115.5 (7)
C9—C14—H14	120.3	F27A—C39—C37	108.5 (10)
C13—C14—C9	119.3 (3)	F28—C40—C35	111.2 (5)
C13—C14—H14	120.3	F28A—C40—F29A	109.6 (9)
F7—C15—F8	102.4 (10)	F28A—C40—F30A	103.3 (8)
F7—C15—F9	105.2 (12)	F28A—C40—C35	118.7 (7)
F7—C15—C13	116.4 (9)	F29—C40—F28	100.5 (8)
F7A—C15—C13	111.2 (7)	F29—C40—F30	111.6 (8)
F8—C15—F9	105.8 (10)	F29—C40—C35	115.9 (7)
F8—C15—C13	117.9 (7)	F29A—C40—F30A	100.7 (8)
F8A—C15—F7A	105.5 (8)	F29A—C40—C35	113.0 (7)
F8A—C15—C13	109.7 (5)	F30—C40—F28	101.7 (7)
F9—C15—C13	108.0 (8)	F30—C40—C35	114.1 (7)
F9A—C15—F7A	106.7 (8)	F30A—C40—C35	109.5 (6)
F9A—C15—F8A	106.8 (7)	C42—C41—O6	118.4 (3)
F9A—C15—C13	116.3 (6)	C42—C41—C46	121.0 (3)
F10—C16—F11	109.5 (9)	C46—C41—O6	120.5 (3)
F10—C16—F12	104.5 (9)	C41—C42—H42	120.4
F10—C16—C11	115.5 (8)	C41—C42—C43	119.3 (3)
F10A—C16—C11	108.7 (7)	C43—C42—H42	120.4
F11—C16—C11	112.2 (5)	C42—C43—C48	118.9 (4)
F11A—C16—F10A	96.3 (7)	C44—C43—C42	120.4 (3)
F11A—C16—C11	115.0 (5)	C44—C43—C48	120.6 (4)
F12—C16—F11	103.7 (8)	C43—C44—H44	120.1
F12—C16—C11	110.5 (6)	C43—C44—C45	119.7 (3)
F12A—C16—F10A	111.6 (11)	C45—C44—H44	120.1
F12A—C16—F11A	108.5 (8)	C44—C45—C46	120.1 (3)
F12A—C16—C11	115.2 (7)	C44—C45—C47	120.6 (3)
C18—C17—O3	123.0 (3)	C46—C45—C47	119.3 (3)
C18—C17—C22	121.9 (3)	C41—C46—C45	119.5 (3)
C22—C17—O3	115.0 (3)	C41—C46—H46	120.3
C17—C18—H18	120.2	C45—C46—H46	120.3
C17—C18—C19	119.5 (3)	F34—C47—F35	106.4 (8)
C19—C18—H18	120.2	F34—C47—C45	114.6 (6)
C18—C19—C24	120.1 (3)	F34A—C47—C45	111.5 (7)
C20—C19—C18	119.5 (4)	F35—C47—C45	114.5 (6)
C20—C19—C24	120.4 (4)	F35A—C47—F34A	106.1 (9)
C19—C20—H20	119.9	F35A—C47—F36A	107.0 (10)
C21—C20—C19	120.3 (3)	F35A—C47—C45	113.2 (7)
C21—C20—H20	119.9	F36—C47—F34	103.5 (6)
C20—C21—C22	120.6 (3)	F36—C47—F35	102.0 (7)
C20—C21—C23	118.9 (4)	F36—C47—C45	114.5 (6)
C22—C21—C23	120.6 (4)	F36A—C47—F34A	108.0 (10)
C17—C22—H22	120.9	F36A—C47—C45	110.7 (8)
C21—C22—C17	118.2 (3)	F31—C48—F32	106.6 (9)
C21—C22—H22	120.9	F31—C48—C43	111.2 (6)
F13—C23—F15	105.5 (9)	F31A—C48—F33A	105.6 (10)
F13—C23—C21	112.8 (7)	F31A—C48—C43	113.4 (9)
F13A—C23—F14A	100.1 (13)	F32—C48—C43	113.6 (7)
F13A—C23—C21	115.9 (13)	F32A—C48—F31A	112.4 (12)
F14—C23—F13	105.2 (9)	F32A—C48—F33A	101.3 (11)
F14—C23—F15	109.1 (9)	F32A—C48—C43	110.0 (10)
F14—C23—C21	110.6 (7)	F33—C48—F31	106.6 (9)
F14A—C23—C21	117.1 (10)	F33—C48—F32	102.3 (8)
F15—C23—C21	113.2 (8)	F33—C48—C43	115.8 (7)
F15A—C23—F13A	105.9 (14)	F33A—C48—C43	113.5 (8)
F15A—C23—F14A	100.9 (14)		
			
P1—O1—C1—C2	−52.2 (3)	C18—C19—C24—F16	0.2 (11)
P1—O1—C1—C6	129.3 (2)	C18—C19—C24—F16A	−46.3 (13)
P1—O2—C9—C10	−154.0 (2)	C18—C19—C24—F17	113.4 (11)
P1—O2—C9—C14	26.6 (4)	C18—C19—C24—F17A	75.8 (11)
P2—O3—C17—C18	39.6 (4)	C18—C19—C24—F18	−121.2 (16)
P2—O3—C17—C22	−143.7 (2)	C18—C19—C24—F18A	−167.2 (10)
P2—O4—C25—C26	−80.0 (3)	C19—C20—C21—C22	1.5 (5)
P2—O4—C25—C30	101.4 (3)	C19—C20—C21—C23	−178.9 (4)
P3—O5—C33—C34	−102.5 (3)	C20—C19—C24—F16	−179.1 (10)
P3—O5—C33—C38	80.5 (3)	C20—C19—C24—F16A	134.4 (13)
P3—O6—C41—C42	−105.7 (3)	C20—C19—C24—F17	−65.9 (11)
P3—O6—C41—C46	78.0 (3)	C20—C19—C24—F17A	−103.5 (11)
O1—P1—O2—C9	50.1 (2)	C20—C19—C24—F18	59.5 (16)
O1—P1—N1—P2	132.43 (16)	C20—C19—C24—F18A	13.5 (11)
O1—P1—N3—P3	−142.11 (15)	C20—C21—C22—C17	−0.8 (5)
O1—C1—C2—C3	−179.8 (3)	C20—C21—C23—F13	179.6 (11)
O1—C1—C6—C5	177.7 (2)	C20—C21—C23—F13A	146 (2)
O2—P1—O1—C1	−141.5 (2)	C20—C21—C23—F14	−62.8 (12)
O2—P1—N1—P2	−116.75 (17)	C20—C21—C23—F14A	−96 (2)
O2—P1—N3—P3	109.95 (17)	C20—C21—C23—F15	59.9 (12)
O2—C9—C10—C11	−178.9 (3)	C20—C21—C23—F15A	22 (3)
O2—C9—C14—C13	179.2 (3)	C22—C17—C18—C19	0.3 (5)
O3—P2—O4—C25	−174.0 (2)	C22—C21—C23—F13	−0.8 (12)
O3—P2—N1—P1	−121.81 (16)	C22—C21—C23—F13A	−34 (2)
O3—P2—N2—P3	129.18 (17)	C22—C21—C23—F14	116.8 (11)
O3—C17—C18—C19	176.8 (3)	C22—C21—C23—F14A	84 (2)
O3—C17—C22—C21	−176.8 (3)	C22—C21—C23—F15	−120.5 (11)
O4—P2—O3—C17	57.9 (3)	C22—C21—C23—F15A	−158 (2)
O4—P2—N1—P1	128.29 (16)	C23—C21—C22—C17	179.6 (4)
O4—P2—N2—P3	−122.54 (17)	C24—C19—C20—C21	178.1 (4)
O4—C25—C26—C27	−178.5 (2)	C25—C26—C27—C28	0.3 (5)
O4—C25—C30—C29	178.4 (3)	C25—C26—C27—C32	179.6 (3)
O5—P3—O6—C41	178.1 (2)	C26—C25—C30—C29	−0.2 (5)
O5—P3—N2—P2	115.28 (17)	C26—C27—C28—C29	−0.6 (5)
O5—P3—N3—P1	−105.67 (17)	C26—C27—C32—F22	172.2 (8)
O5—C33—C34—C35	−177.6 (3)	C26—C27—C32—F22A	−147.2 (17)
O5—C33—C38—C37	177.9 (3)	C26—C27—C32—F23	51.5 (12)
O6—P3—O5—C33	70.5 (2)	C26—C27—C32—F23A	93.2 (12)
O6—P3—N2—P2	−137.14 (16)	C26—C27—C32—F24	−68.2 (10)
O6—P3—N3—P1	145.21 (15)	C26—C27—C32—F24A	−20.5 (14)
O6—C41—C42—C43	−175.0 (3)	C27—C28—C29—C30	0.6 (5)
O6—C41—C46—C45	176.4 (2)	C27—C28—C29—C31	179.3 (4)
N1—P1—O1—C1	−23.1 (3)	C28—C27—C32—F22	−8.5 (10)
N1—P1—O2—C9	−65.7 (2)	C28—C27—C32—F22A	32.1 (18)
N1—P1—N3—P3	−15.8 (2)	C28—C27—C32—F23	−129.2 (11)
N1—P2—O3—C17	−56.6 (3)	C28—C27—C32—F23A	−87.5 (12)
N1—P2—O4—C25	−55.4 (2)	C28—C27—C32—F24	111.0 (10)
N1—P2—N2—P3	2.2 (2)	C28—C27—C32—F24A	158.8 (13)
N2—P2—O3—C17	174.4 (2)	C28—C29—C30—C25	−0.1 (5)
N2—P2—O4—C25	74.2 (2)	C28—C29—C31—F19	−149.3 (15)
N2—P2—N1—P1	1.4 (2)	C28—C29—C31—F19A	161.8 (9)
N2—P3—O5—C33	−174.2 (2)	C28—C29—C31—F20	−25.6 (11)
N2—P3—O6—C41	65.6 (2)	C28—C29—C31—F20A	−82.8 (13)
N2—P3—N3—P1	19.2 (2)	C28—C29—C31—F21	87.4 (10)
N3—P1—O1—C1	107.9 (2)	C28—C29—C31—F21A	38.3 (17)
N3—P1—O2—C9	165.2 (2)	C30—C25—C26—C27	0.1 (4)
N3—P1—N1—P2	5.3 (2)	C30—C29—C31—F19	29.4 (16)
N3—P3—O5—C33	−44.0 (3)	C30—C29—C31—F19A	−19.4 (11)
N3—P3—O6—C41	−64.1 (2)	C30—C29—C31—F20	153.1 (9)
N3—P3—N2—P2	−12.3 (2)	C30—C29—C31—F20A	95.9 (13)
C1—C2—C3—C4	2.2 (5)	C30—C29—C31—F21	−93.9 (10)
C1—C2—C3—C7	−178.5 (3)	C30—C29—C31—F21A	−143.0 (16)
C2—C1—C6—C5	−0.9 (4)	C31—C29—C30—C25	−178.9 (4)
C2—C3—C4—C5	−0.9 (5)	C32—C27—C28—C29	−179.9 (4)
C2—C3—C7—F1	7.7 (8)	C33—C34—C35—C36	−0.3 (5)
C2—C3—C7—F1A	39 (2)	C33—C34—C35—C40	179.0 (3)
C2—C3—C7—F2	−114.9 (10)	C34—C33—C38—C37	1.0 (5)
C2—C3—C7—F2A	−80.7 (16)	C34—C35—C36—C37	0.8 (5)
C2—C3—C7—F3	125.8 (9)	C34—C35—C40—F28	−37.2 (9)
C2—C3—C7—F3A	162 (2)	C34—C35—C40—F28A	−100.3 (13)
C3—C4—C5—C6	−1.3 (5)	C34—C35—C40—F29	76.8 (10)
C3—C4—C5—C8	178.9 (3)	C34—C35—C40—F29A	30.1 (12)
C4—C3—C7—F1	−172.9 (7)	C34—C35—C40—F30	−151.5 (10)
C4—C3—C7—F1A	−141 (2)	C34—C35—C40—F30A	141.4 (9)
C4—C3—C7—F2	64.5 (11)	C35—C36—C37—C38	−0.5 (5)
C4—C3—C7—F2A	98.7 (16)	C35—C36—C37—C39	178.5 (4)
C4—C3—C7—F3	−54.8 (10)	C36—C35—C40—F28	142.0 (8)
C4—C3—C7—F3A	−19 (2)	C36—C35—C40—F28A	78.9 (14)
C4—C5—C6—C1	2.2 (4)	C36—C35—C40—F29	−104.0 (10)
C4—C5—C8—F4	143.7 (12)	C36—C35—C40—F29A	−150.7 (11)
C4—C5—C8—F4A	105.6 (13)	C36—C35—C40—F30	27.7 (11)
C4—C5—C8—F5	22.5 (14)	C36—C35—C40—F30A	−39.3 (10)
C4—C5—C8—F5A	−13.5 (12)	C36—C37—C38—C33	−0.4 (5)
C4—C5—C8—F6	−102.6 (8)	C36—C37—C39—F25	−77.3 (11)
C4—C5—C8—F6A	−134.2 (16)	C36—C37—C39—F25A	−53.0 (17)
C6—C1—C2—C3	−1.3 (4)	C36—C37—C39—F26	164.1 (10)
C6—C5—C8—F4	−36.1 (12)	C36—C37—C39—F26A	−174.1 (18)
C6—C5—C8—F4A	−74.2 (13)	C36—C37—C39—F27	42.6 (13)
C6—C5—C8—F5	−157.3 (13)	C36—C37—C39—F27A	62.6 (15)
C6—C5—C8—F5A	166.7 (11)	C38—C33—C34—C35	−0.6 (4)
C6—C5—C8—F6	77.6 (9)	C38—C37—C39—F25	101.7 (11)
C6—C5—C8—F6A	46.0 (16)	C38—C37—C39—F25A	125.9 (17)
C7—C3—C4—C5	179.8 (3)	C38—C37—C39—F26	−16.9 (11)
C8—C5—C6—C1	−178.0 (3)	C38—C37—C39—F26A	4.8 (19)
C9—C10—C11—C12	0.3 (5)	C38—C37—C39—F27	−138.5 (12)
C9—C10—C11—C16	179.5 (4)	C38—C37—C39—F27A	−118.5 (15)
C10—C9—C14—C13	−0.1 (5)	C39—C37—C38—C33	−179.4 (3)
C10—C11—C12—C13	−1.4 (5)	C40—C35—C36—C37	−178.5 (4)
C10—C11—C16—F10	−85.5 (12)	C41—C42—C43—C44	−1.8 (5)
C10—C11—C16—F10A	−125.4 (8)	C41—C42—C43—C48	176.6 (4)
C10—C11—C16—F11	41.0 (12)	C42—C41—C46—C45	0.2 (4)
C10—C11—C16—F11A	−18.8 (10)	C42—C43—C44—C45	0.7 (5)
C10—C11—C16—F12	156.2 (7)	C42—C43—C48—F31	−78.3 (13)
C10—C11—C16—F12A	108.6 (14)	C42—C43—C48—F31A	−150.2 (16)
C11—C12—C13—C14	1.7 (5)	C42—C43—C48—F32	161.5 (8)
C11—C12—C13—C15	−178.8 (4)	C42—C43—C48—F32A	82.9 (17)
C12—C11—C16—F10	93.8 (12)	C42—C43—C48—F33	43.5 (11)
C12—C11—C16—F10A	53.9 (9)	C42—C43—C48—F33A	−29.8 (13)
C12—C11—C16—F11	−139.7 (11)	C43—C44—C45—C46	0.8 (5)
C12—C11—C16—F11A	160.5 (8)	C43—C44—C45—C47	−178.6 (3)
C12—C11—C16—F12	−24.6 (9)	C44—C43—C48—F31	100.1 (13)
C12—C11—C16—F12A	−72.2 (15)	C44—C43—C48—F31A	28.2 (17)
C12—C13—C14—C9	−1.0 (5)	C44—C43—C48—F32	−20.1 (10)
C12—C13—C15—F7	−178.5 (15)	C44—C43—C48—F32A	−98.7 (17)
C12—C13—C15—F7A	−140.9 (9)	C44—C43—C48—F33	−138.1 (9)
C12—C13—C15—F8	59.2 (14)	C44—C43—C48—F33A	148.7 (12)
C12—C13—C15—F8A	102.8 (10)	C44—C45—C46—C41	−1.3 (4)
C12—C13—C15—F9	−60.6 (15)	C44—C45—C47—F34	−131.5 (8)
C12—C13—C15—F9A	−18.5 (11)	C44—C45—C47—F34A	−170.2 (13)
C14—C9—C10—C11	0.5 (5)	C44—C45—C47—F35	−8.2 (12)
C14—C13—C15—F7	1.0 (16)	C44—C45—C47—F35A	−50.7 (10)
C14—C13—C15—F7A	38.6 (10)	C44—C45—C47—F36	109.1 (7)
C14—C13—C15—F8	−121.3 (13)	C44—C45—C47—F36A	69.5 (14)
C14—C13—C15—F8A	−77.7 (10)	C46—C41—C42—C43	1.3 (4)
C14—C13—C15—F9	118.9 (14)	C46—C45—C47—F34	49.1 (9)
C14—C13—C15—F9A	161.0 (9)	C46—C45—C47—F34A	10.4 (13)
C15—C13—C14—C9	179.5 (4)	C46—C45—C47—F35	172.4 (11)
C16—C11—C12—C13	179.4 (4)	C46—C45—C47—F35A	129.9 (9)
C17—C18—C19—C20	0.3 (5)	C46—C45—C47—F36	−70.3 (8)
C17—C18—C19—C24	−179.0 (4)	C46—C45—C47—F36A	−109.9 (14)
C18—C17—C22—C21	0.0 (5)	C47—C45—C46—C41	178.1 (3)
C18—C19—C20—C21	−1.2 (5)	C48—C43—C44—C45	−177.7 (4)

### Hexakis[3,5-bis(trifluoromethyl)phenoxy]cyclotriphosphazene (3) Hydrogen-bond geometry (Å, º)

**Table:**

*D*—H···*A*	*D*—H	H···*A*	*D*···*A*	*D*—H···*A*
C10—H10···F26	0.93	2.58	3.478 (10)	163
C30—H30···F6*A*^i^	0.93	2.69	3.379 (14)	132
C36—H36···F32*A*^ii^	0.93	2.59	3.40 (2)	146
C44—H44···F29^iii^	0.93	2.67	3.521 (13)	152

Symmetry codes: (i) *x*+1, *y*, *z*; (ii) *x*, *y*+1, *z*; (iii) −*x*+1, −*y*+1, −*z*.
